# Durable radiative cooling against environmental aging

**DOI:** 10.1038/s41467-022-32409-7

**Published:** 2022-08-16

**Authors:** Jianing Song, Wenluan Zhang, Zhengnan Sun, Mengyao Pan, Feng Tian, Xiuhong Li, Ming Ye, Xu Deng

**Affiliations:** 1 Institute of Fundamental and Frontier Sciences, University of Electronic Science and Technology of China, Chengdu, 610054 China; 2 School of Automation Engineering, University of Electronic Science and Technology of China, Chengdu, 611731 China; 3 Shanghai Synchrotron Radiation Facility, Zhangjiang Lab, Shanghai Advanced Research Institute, Chinese Academy of Science, Shanghai, 201204 China; 4 Material Physics and Analytics, BASF Advanced Chemicals Co., Ltd. 333 Jiang Xin Sha Road, Pudong, Shanghai, 200137 China; 5 Shenzhen Institute for Advanced Study, University of Electronic Science and Technology of China, Shenzhen, 518110 China

**Keywords:** Mechanical engineering, Surfaces, interfaces and thin films

## Abstract

To fight against global warming, subambient daytime radiative cooling technology provides a promising path to meet sustainable development goals. To achieve subambient daytime radiative cooling, the reflection of most sunlight is the essential prerequisite. However, the desired high solar reflectance is easily dampened by environmental aging, mainly natural soiling and ultraviolet irradiation from sunlight causing yellowish color for most polymers, making the cooling ineffective. We demonstrate a simple strategy to use titanium dioxide nanoparticles, with ultraviolet resistance, forming hierarchical porous morphology via evaporation-driven assembly, which guarantees a balanced anti-soiling and high solar reflectance, rendering anti-aging cooling paint based coatings. We challenge the cooling coatings in an accelerated weathering test against simulated 3 years of natural soiling and simulated 1 year of natural sunshine, and find that the solar reflectance only declined by 0.4% and 0.5% compared with the un-aged ones. We further show over 6 months of aging under real-world conditions with barely no degradation to the cooling performance. Our anti-aging cooling paint is scalable and can be spray coated on desired outdoor architecture and container, presenting durable radiative cooling, promising for real-world applications.

## Abstract (web summary)

The high solar reflectance needed by radiative cooling is easily dampened by environmental aging. Here, authors describe durable cooling performance against heavy soiling and long-term ultraviolet exposure of paint-based coatings, enhancing the potential of radiative cooling for real-world applications.

## Introduction

Our world is going awry to achieve the 1.5 °C objective of the Paris Agreement because the global greenhouse gas (GHG) emissions are still soaring^1^. If no urgent action is taken to mitigate the GHG emissions, some parts of the world will be almost unlivable for human beings by the end of this century^2^. Nowadays, >10% of GHG emissions is from conventional space cooling and refrigeration^3^. As the world becomes warmer, >13 new cooling devices are installed every second globally, which creates more GHG emissions and ozone-depleting substances exacerbating the global warming^4^. To fight against this destructive feedback loop, subambient daytime radiative cooling (SDRC) technology provides a promising path. It is realized by a sky-facing object reflecting most sunlight (within wavelength of 0.3 to 2.5 µm) and emitting long-wave infrared (LWIR) radiation strongly to the cold universe through the atmospheric transparency window (within wavelength of 8–13 µm)^5–21^. In this manner, the object can be passively cooled below the ambient temperature with zero energy input and GHG emissions.

**Fig. 1:**
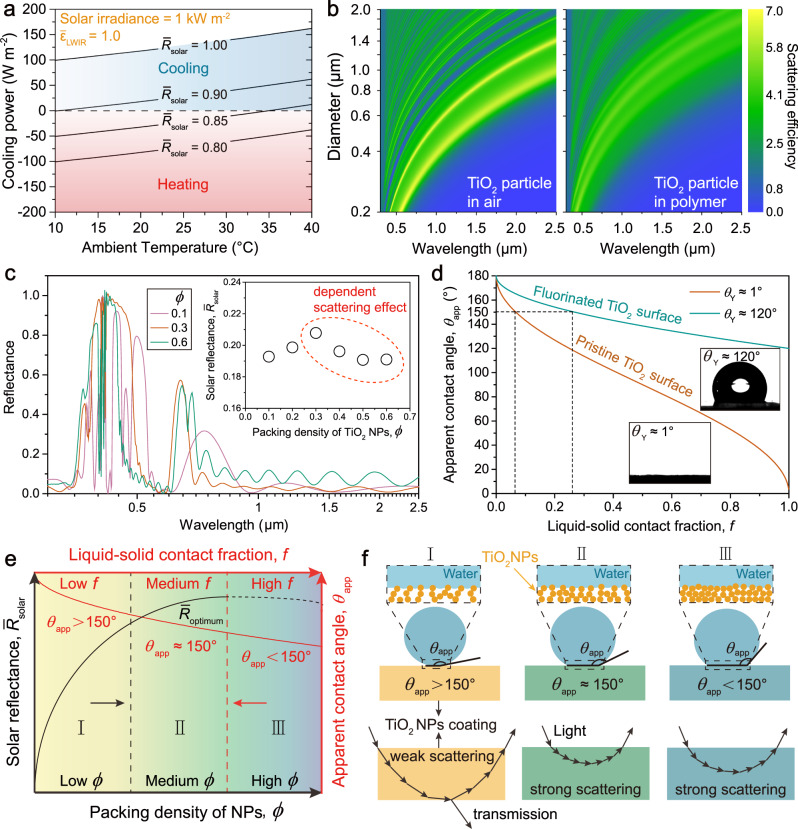
Design of AACP. **a** Net cooling power as a function of ambient temperature for various *R̅*_solar_. Theoretical calculation is based on steady state heat transfer balance analysis (details in Method). Subambient cooling is difficult to achieve when *R̅*_solar_ < 0.9 even if the material has a perfect ${\bar{\varepsilon }}_{{{{{{\rm{LWIR}}}}}}}$. **b** The scattering efficiency of a single TiO_2_ spherical particle as a function of particle diameter over wavelength range of 0.3 to 2.5 µm in different scattering mediums. **c** Simulated reflectance curves and resulted *R̅*_solar_ values as a function of TiO_2_ nanoparticles (NPs) packing density (*ϕ*) for a film of 2 µm thickness. The declining trend of reflectance in the dashed circle is due to the dependent scattering effect from particle crowding. **d** Relationship between apparent water contact angle (*θ*_app_*)* and the intrinsic Young’s contact angle (*θ*_Y_) of TiO_2_ surfaces. **e** Anticipated *R̅*_solar_ and *θ*_app_ as a function of *ϕ* resulting from **f** the three proposed regimes: I, II, and III. A trade-off between the *R̅*_solar_ and *θ*_app_ renders regime II as the design target to obtain excellent radiative cooling and anti-soiling performances at the same time. The degree of dependent scattering adds complexity to anticipate the variation of *R̅*_solar_ when *ϕ* is high. Therefore, in **e**, the dashed line of the solar reflectance data curve only indicates a possible variation trend.

The core of SDRC lies in the high solar reflectance (*R̅*_solar_ ≥ 0.9), since just a few percent of solar absorbance can effectively heat the surface even if it has perfect LWIR emittance, i.e. ${\bar{\varepsilon }}_{{{{{{\rm{LWIR}}}}}}}$ = 1 (Fig. 1a). However, this indispensable high *R̅*_solar_ is very likely to decline, making this technology ineffective, after the SDRC materials exposed to outdoor natural environment for just several months, which is essentially resulted from natural environmental aging^22^. Although SDRC ability in the ideal scenarios have been demonstrated by diverse materials, like nanophotonic thin films^5^, polymer-dielectric composites backed with metal mirrors^7^, polymeric nano-textiles^19,23,24^, nanocellulose^11^ and porous polymer coatings^9,17,18^, these materials were seldom evaluated against environmental aging, mainly natural soiling and UV irradiation from sunlight^22^. Among them, most polymers for SDRC, even if not consider the darkening effect caused by natural soiling, are not resistant to long-term UV exposure, which results in yellowish appearance lowering the *R̅*_solar_^25^. While porous fluoropolymer based coating for SDRC is resistant to UV^9,26^, its modest hydrophobic nature limits the anti-soiling performance^27^. Therefore, to push SDRC towards real-world applications with long-term durability, besides exceptional optical properties, excellent soiling/UV resistances are highly desired, preferred together with ease of fabrication and scalability in the form of paint based coatings.

In this work, in response to above daunting requirements, we demonstrate anti-aging cooling paint (AACP) based coating, which features evaporation-driven assembled hierarchical porous morphology, composed by hydrophobic titanium dioxide (TiO_2_) nanoparticles (NPs). They exhibit excellent cooling abilities even after challenged against accelerated weathering tests of simulated 3 years of natural soiling, simulated 1 year of natural sunshine, and over 6 months of aging under real-world conditions. Our AACP is a suspension that can be sprayed and painted on diverse substrates in a scalable manner, promising for commercial applications.

## Results

### Design of AACP

We designed the AACP based on three criteria: (1) the material must be intrinsically resistant to UV irradiance, (2) the derived coating must have *R̅*_solar_ ≥ 0.9 and (3) the coating surface must be able to reduce the accretion of substances (e.g. common soiling contaminants). To satisfy the first criterion, we chose not to use polymer binders in our formulation, because most common polymers are reactive to UV light, become yellowish after long-term UV exposure^25^. Instead, we used rutile type TiO_2_ NPs to form porous coating layers. TiO_2_ NPs are strong light scatters (refractive index ${n}_{{{{{{\rm{Ti}}}}}}{{{{{{\rm{O}}}}}}}_{2}}$ ≈ 2.7), commonly used as UV mitigation component, commercially available and cost effective^28^. Although TiO_2_ has been studied for daytime radiative cooling since 1970s, the lack of ability to realize subambient cooling under direct sunlight makes its practical value very limited^29^. Subambient radiative cooling under direct sunlight based on TiO_2_ has been realized only recently by adding fluorescent particles for enhanced *R̅*_solar_ and silica microspheres for higher ${\bar{\varepsilon }}_{{{{{{\rm{LWIR}}}}}}}$^30^, which creates TiO_2_ based radiative cooling paint of more complexity. Conventionally the UV absorptance of TiO_2_ NPs and near-infrared (NIR) absorptance of common polymer binders were believed to have adverse impact for paint based coatings to achieve *R̅*_solar_ > 0.9^31^. However, we note that the energy proportion within UV region (wavelength of 0.28 to 0.4 µm) only accounts for 4.5% of the whole solar spectrum (Supplementary Fig. 1). Therefore, we can compensate the UV absorptance via suppressing the NIR absorptance by replacing polymer with air forming porous morphology. And we note that the refractive index of air (${n}_{{{{{{\rm{air}}}}}}}$ = 1) is lower than the one of common polymer binder (${n}_{{{{{{\rm{polym}}}}}}}$ ≈ 1.5)^32^. According to the Snell’s law, large refractive index difference between two different mediums leads to high magnitude of light refraction. Thus, TiO_2_ NPs in air should scatter light more strongly than they do in polymers. To further evaluate this optical property theoretically, we numerically calculated the scattering efficiency (*Q*_sca_) of a single spherical TiO_2_ particle as a function of particle diameter across the solar spectrum based on Mie theory (details in Methods). We compared the *Q*_sca_ of TiO_2_ particle in surrounding medium of either air or polymer with preset refractive indices (${n}_{{{{{{\rm{air}}}}}}}$ = 1 and ${n}_{{{{{{\rm{polym}}}}}}}$ = 1.5). We found that, within the solar spectrum, a single TiO_2_ particle could scatter sunlight more strongly in air than in polymer (Fig. 1b). Empirically, the magnitude of *R̅*_solar_ is equivalent to the total magnitude of TiO_2_ NPs scattering, which is determined by the number of air/particle interfaces the light passes through. Therefore, for coatings with equal thickness composed of TiO_2_ NPs with the same size, high packing density (*ϕ*) of NPs, which means large number of air/particle interfaces, should be able to satisfy the second requirement (*R̅*_solar_ ≥ 0.9). However, we note that the crowding of TiO_2_ NPs gives rise to the dependent scattering leading to a reduction of scattering efficiency, in contrast to independent scattering wherein the distance among the scattering particles large enough to ignore the scattering effect brought by the presence of neighboring particles^33^. This phenomenon is evident for a thin coating composed of NPs with high packing density, corroborated by finite-difference time-domain (FDTD) simulations (Fig. 1c). In practice, to compensate this adverse dependent scattering effect, we can fabricate thick coating composed of particles with broad size distribution to increase the total scattering power (Supplementary Fig. 5). Nevertheless, continual increasing the *ϕ* of TiO_2_ NPs might not render higher *R̅*_solar_ as expected intuitively. In another word, we should be able to obtain high *R̅*_solar_ with *ϕ* lying in the medium region, neither very low nor high.

To meet the last criterion of the ability to reduce the accretion of substances, we aim to obtain low surface adhesion force by means of non-wettability^27,34,35^. Herein, the governing equation is based on the Cassie-Baxter model (Supplementary Figs. 2, 10 and 11), i.e. cos *θ*_app_ = *f* (1 + cos *θ*_Y_) −1, where *θ*_app_ is the apparent contact angle, *θ*_Y_ is the Young’s contact angle, and *f* the liquid-solid contact fraction (details in Methods). As shown in Fig. 1d, *θ*_Y_ of pristine flat smooth TiO_2_ surface is only ~1°. To achieve the critical value for non-wetting surface, 150° of *θ*_app_, it is required that *f*  < 10%, which is very challenging for scalable practical application. Fluorinated flat smooth TiO_2_ surface has *θ*_Y_ as ~120°, then the critical value of *f* increases to ~0.3 to have *θ*_app_ ≥ 150° to realize non-wettability, making porous surface topology a potential candidate.

Previously, non-wettability was realized by constructing hierarchical surface morphology from the random packed fluorinated TiO_2_ NPs^36^. However, solely tuning the surface morphology cannot meet our target, which is realizing the above three criteria at the same time: resistant to UV irradiation, high *R̅*_solar_ and great non-wettability. Hence, besides surface morphology, bulk morphology and its associated optical and wetting properties must be considered altogether to obtain durable radiative cooling performance. We consider the coating’s surface and bulk owning similar morphology, thereby the magnitude of *f* should be in direct proportion to the magnitude of *ϕ* (Supplementary Fig. 6). Hence, from the perspective of qualitative analysis, we propose that the magnitude of *ϕ* and *f* leads to three distinct regimes, as shown in the schematic Fig. 1e, and anticipate *R̅*_solar_ and *θ*_app_ to variate as a function of TiO_2_ NPs packing density (*ϕ*) resulting from these three regimes. Each regime features a characteristic set of *R̅*_solar_ and *θ*_app_, corresponding to schematic presented in Fig. 1f, with hypothesized light path and liquid-solid contact interface. In regime I, the low *ϕ* leads to relatively weak light scattering, thus the light has a long path length, penetrates deeply into the coating, may reach the substrate, resulting in light transmission. While at the same time, the low *f* leads to *θ*_app_ > 150°, indicating good non-wettability. In regime III, the scenario is just the opposite. The high *ϕ* leads to strong light scattering, a short path length, thus shallow penetration, which turns the light around relatively quickly. The high *f* leads to *θ*_app_ < 150° indicating modest hydrophobicity, not beneficial to anti-soiling purpose. Therefore, regime II is preferred to be the design target to balance the desired high *R̅*_solar_ and *θ*_app_.

### Fabrication of AACP based coatings and their cooling performances

**Fig. 2:**
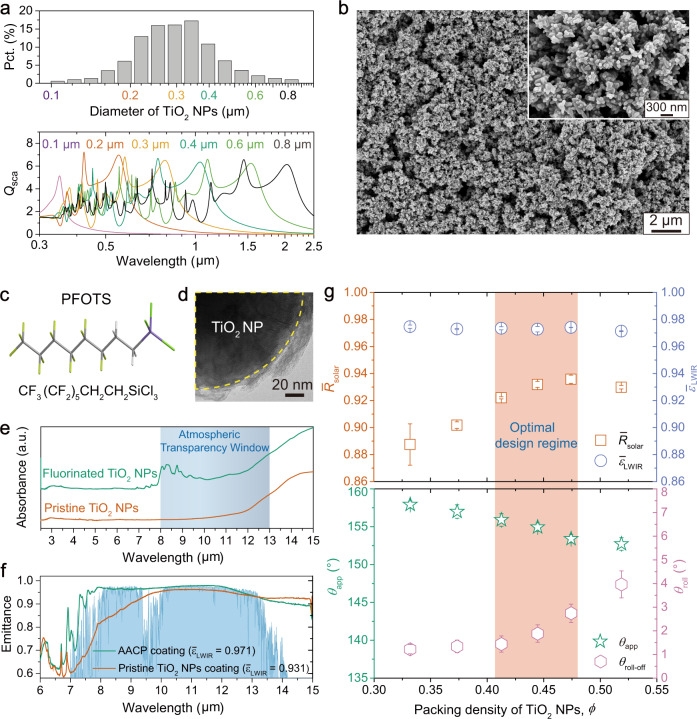
The optical and wetting properties of AACP. **a** TiO_2_ NPs size distribution (characterized by dynamic light scattering) and the calculated scattering efficiency (*Q*_sca_) as a function of particle size and incident light wavelength. The strong scattering peaks from particles with various sizes cover the whole solar spectrum in favor of high solar reflectance (*R̅*_solar_). **b** Scanning electron microscopy image showing the AACP coating surface composed of TiO_2_ NPs forming micro/nano-roughness. **c** A wireframe showing the structure of PFOTS. **d** TEM image showing the fluoride component grafted on the surface of TiO_2_ NP. **e** Fourier transform infrared absorbance spectrum showing the C−F stretching vibration between 7.35 to 10 µm due to fluorination, leading to **f** high emittance of AACP coating in the corresponding wavelength range. **g** *R̅*_solar_, ${\bar{\varepsilon }}_{{{{{{\rm{LWIR}}}}}}}$, *θ*_app_ and *θ*_roll_ as a function of TiO_2_ NPs packing density (*ϕ*). The shaded area represents a range of *ϕ* for optimal optical and wetting properties.

From these principles, we created AACP by selecting TiO_2_ NPs with size distribution centering around 0.3 µm. This wide size distribution is not only useful for TiO_2_ NPs to strongly scatter light within the solar spectrum through collective effect of Mie resonances^24^ (Fig. 2a, Supplementary Fig. 5), but also helpful to obtain micro/nano-roughness for robust non-wettability^36^. We mixed the TiO_2_ NPs and perfluorooctyltrichlorosilane (PFOTS) into ethanol to create an AACP suspension. We dispensed this suspension in drops onto the substrate, let the ethanol evaporate to form a uniform coating layer (details in Methods). As presented in Fig. 2b, the coating shows TiO_2_ NPs assembles at dual length scales forming micro/nano-roughness. Ultra-small X-ray scattering (USAXS) was implemented to characterize the coating’s bulk morphology demonstrating hierarchical porous nature, featuring air pores with two distinct length scales at ~110 and 350 nm (details in Methods) (Supplementary Fig. 7). PFOTS (Fig. 2c) was grafted on the surface of TiO_2_ NPs through silanization, shown by transmission electron microscopy (TEM) as a blurred outer layer (Fig. 2d). The silanization reaction not only adds fluoride component lowering the surface energy of the AACP coating, but also enhances its emittance within atmospheric transparency window, because of the collective stretching vibrations from carbon-fluorine (C − F), carbon-carbon (C − C), silicon-oxygen (Si − O) and silicon-oxygen-silicon (Si − O − Si) bonds between 7 and 10 µm (Fig. 2e and f, and Supplementary Fig. 8)^37^. We evaluated the AACP coating’s optical and wetting properties as a function of TiO_2_ NPs packing density (*ϕ* from 0.33 to 0.53), presented in Fig. 2g. *R̅*_solar_ climbs to ~0.94 with dense packing of TiO_2_ NPs, while ${\bar{\varepsilon }}_{{{{{{\rm{LWIR}}}}}}}$ (~0.97) is insensitive to the variation of *ϕ*. Since the variation of *θ*_app_, decreasing from 158° to 153°, is not significant compared to the large contact angles, we measured the roll-off angle (*θ*_roll_) as a complement^38^, showing a rising trend from 1.2° to 4.0°. We note that these results agree with our anticipations as shown in Fig. 1e, rendering a range of *ϕ* centering around 0.45 for optimal optical and wetting properties. Specifically, with *ϕ* ≈ 0.45, we are able to routinely fabricate super-repellent AACP coating with *R̅*_solar_ = 0.93 ± 0.02 and ${\bar{\varepsilon }}_{{{{{{\rm{LWIR}}}}}}}$ = 0.97 ± 0.01 in a highly reproducible way. And we realized these excellent optical properties with coating thickness of only about 100 µm, which is much thinner than the polymeric SDRC coating usually >300 µm^9^ (Supplementary Fig. 9). By using the step-wise heating method, we obtained the cooling power of 84.9 ± 14.8 W m^−2^ under strong solar irradiance (*I*_solar_) of 920 W m^−2^ (Supplementary Fig. 13). And by using the close-tracking heating method, we obtained the cooling power of about 95 W m^−2^ under strong sunshine from 11 AM to 4 PM (Supplementary Fig. 14).

### Standard simulated anti-aging test

**Fig. 3:**
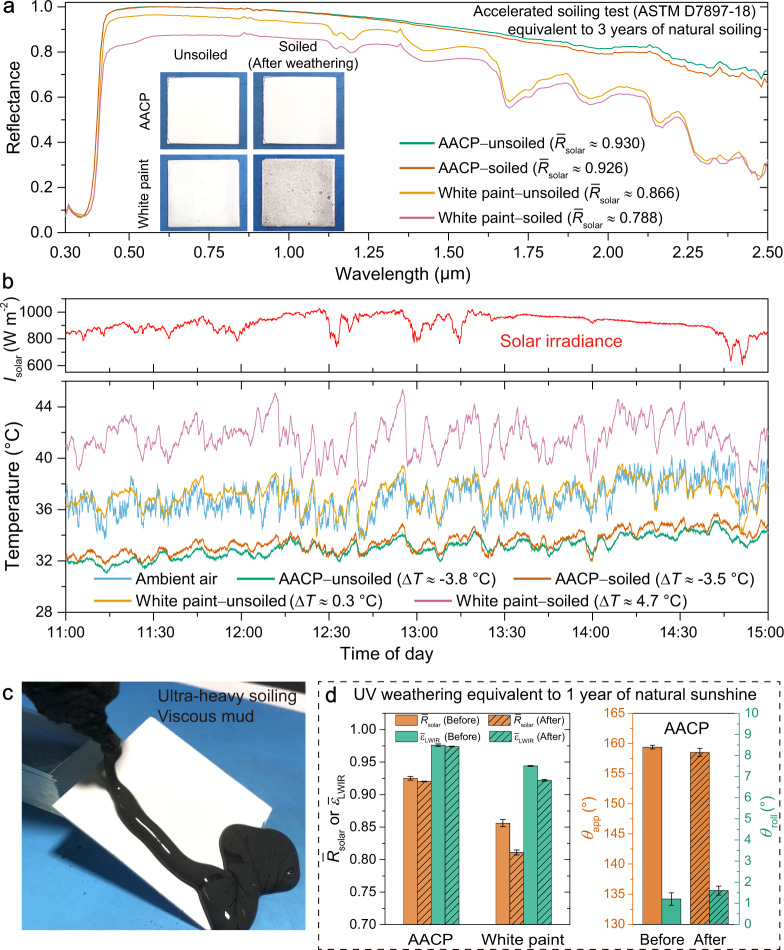
Anti-aging performances of AACP against heavy soiling and long-term UV exposure. **a** *R̅*_solar_ after accelerated soiling test based on ASTM D7897-18 standard, equivalent to 3 years of natural soiling. The soiled AACP coating demonstrated almost intact *R̅*_solar_ compared with the unsoiled one. Insets present visual appearances of the coating samples before and after soiling. **b** Measurements of coating temperature showing excellent anti-soiling performance of the AACP coating (August 23rd, 2021, Chengdu). **c** Viscous mud dripped and flowed off the AACP coating showing excellent self-cleaning function. **d** The almost unchanged optical and wetting properties of AACP coating after 1000 h of UV exposure (0.89 W m^−2^ at 340 nm and 60 °C), equivalent to 1 year of Florida natural sunshine.

One of the most rewarded applications of AACP is believed to be used as cool roofing materials. The real-world aging of cool roofing materials mainly includes soiling and weathering. Soiling mainly results from the deposition of airborne carbon black, dust, particulate organic matter (POM) and microbial growth. Weathering is mainly from exposure to UV irradiation of natural sunlight, accompanied by moisture and temperature variation of day and night. It takes quite a long time, usually several years, for the roofing materials to reach a quasi-steady state of *R̅*_solar_ and ${\bar{\varepsilon }}_{{{{{{\rm{LWIR}}}}}}}$^39^. Hence, to quantitatively evaluate the anti-aging performances of the AACP, we performed accelerated soiling and weathering tests based on the ASTM D7897-18 standard (details in Methods), which was developed particularly to simulate natural environmental aging effects on *R̅*_solar_ and ${\bar{\varepsilon }}_{{{{{{\rm{LWIR}}}}}}}$ of roofing materials in an accelerated way, equivalent to 3 years of natural soiling and 1 year of Florida natural sunshine^40^. Following this standard (Supplementary Fig. 16), we soiled (mixture of soot, dust, POM and salts) and weathered (4 h of simulated rain at 50 °C, 8 h of UV with 0.89 W m^−2^ at 340 nm and 60 °C) the coating samples and then measured the *R̅*_solar_. We found that the *R̅*_solar_ of AACP coating declined only by 0.4% compared with the unsoiled one (from 0.930 to 0.926). While as a comparison, the *R̅*_solar_ of high reflective white paint coating declined by 9.0% (from 0.866 to 0.788, Fig. 3a). The decrease of *R̅*_solar_ for white paint may seem acceptable. However, as we pointed out earlier (see Fig. 1a), to realize SDRC, *R̅*_solar_ needs to be ≥0.9, which is evident in the temperature measurements of these coating samples under strong *I*_solar_ of 915 W m^−2^ (Fig. 3b, peak *I*_solar_ > 1000 W m^−2^, Supplementary Figs. 17 and 18). To mimic real-world operating condition, all field tests were performed without wind shield cover. We recorded the temperature difference (Δ*T*) between the coating sample (*T*_samp_) and ambient air (*T*_air_), where Δ*T* = *T*_samp_ − *T*_air_. The Δ*T* of white paint coating increased from 0.3 to 4.7 °C after soiling. Even the porous fluoropolymer coating, as the state-of-the-art SDRC material, cannot retain its cooling ability against this soiling test, due to the modest hydrophobicity (Supplementary Figs. 19 and 20). Meanwhile, the $\triangle T$ of our AACP coating increased just from −3.8 to −3.5 °C, barely affecting the cooling performance. Additionally, we dripped viscous mud, as ultra-heavy soiling agent, onto the AACP coating to show its excellent ability to reduce the accretion of soiling substances (Fig. 3c, Supplementary Fig. 21, Supplementary Movie 1). The accelerated weathering test was performed by 1000 h of UV exposure at 60 °C (Fig. 3d), equivalent to 1 year of Florida natural sunshine^39^. Owing to the UV resistance of TiO_2_ NPs and strong C − F bonds in PFOTS, the *R̅*_solar_ of AACP coating only declined by 0.5% of the original one (from 0.925 to 0.920). The ${\bar{\varepsilon }}_{{{{{{\rm{LWIR}}}}}}}$, *θ*_app_ and *θ*_roll_ were almost unchanged. As a comparison, the *R̅*_solar_ of white paint coating declined by 5.4% (from 0.856 to 0.810), the ${\bar{\varepsilon }}_{{{{{{\rm{LWIR}}}}}}}$ declined by 2.3% (from 0.944 to 0.922) (Supplementary Fig. 23).

### Real-world test

**Fig. 4:**
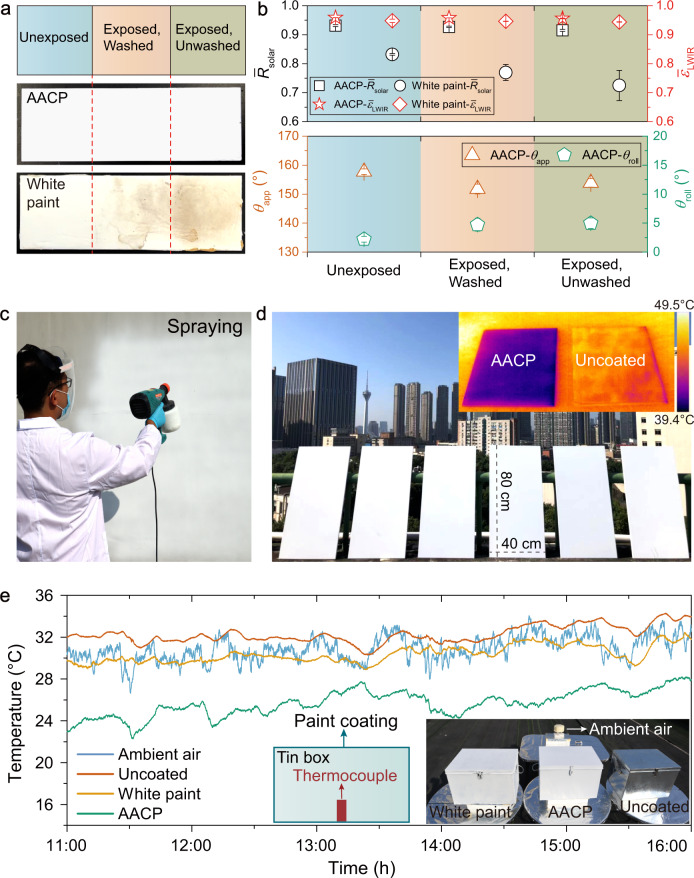
The real-world outdoor durability test, demonstration of versatility, scalability and proof-of-concept. **a** Photographs and **b** the optical and wetting properties of the AACP and white paint coating after 6 months of outdoor exposure (Chengdu, 30°40'36''N, 104°6'28''E, from April 25th to November 10th 2021). We studied the optical and wetting properties of the coatings against environmental aging by dividing the coating slide (5 × 15 cm) into three regions: unexposed/(exposed, washed)/(exposed, unwashed). **c** Demonstration of spray coating and **d** AACP coated outdoor wall tiles (40 × 80 cm). Inset is an infrared image showing the temperature drop of the AACP coated tile (October 29th, 2021, Guangzhou, China). **e** AACP coated tin box (47 × 32 × 32 cm) served as a proof-of-concept, demonstrating SDRC. The averaged *I*_solar_ was 945 W m^−2^ (peak *I*_solar_ > 1000 W m^−2^). Insets show the temperature measurement setup and visual appearance of the tin boxes. For AACP coated tin box, Δ*T*  ≈ −5.2 °C (May 25th, 2021, Chengdu).

Different from simulated aging, the real-world aging is more complex. The soiling and weathering are synergistic to promote the course of aging^39^. To make up for the limitation of simulated aging, we performed over 6 months long outdoor exposure in Chengdu (humid and hot climate) and Xi’an (dry and hot climate) of China to test the durability of the coatings against real-world aging (Fig. 4a, b, and Supplementary Fig. 25). All coating slides were split into three regions: unexposed/(exposed, washed)/(exposed, unwashed). The ${\bar{\varepsilon }}_{{{{{{\rm{LWIR}}}}}}}$ of all samples only had little decrease. For white paint coating, the *R̅*_solar_ of the three regions were 0.832/0.769/0.725, showing a decrease of 7.6% and 12.9% respectively. Remarkably, the decrease of our AACP was only 0.4% and 1.7% (0.931/0.927/0.915). As for self-cleaning properties of AACP coatings, after aging, the *θ*_app_ stayed above 150° and *θ*_roll_ stayed below 5°.

### Mechanical stability and scalability

We further reinforced the mechanical stability of the AACP coatings by utilizing “paint + adhesive” strategy (Supplementary Fig. 26)^36,41^. A layer of adhesive strongly bonds the AACP coating onto the substrate, rendering robustness against high-speed water jet, tape-peel, sand falling abrasion and scratch test (Supplementary Figs. 27–30, Supplementary Table 2, and Supplementary Movie 3). Our AACP formulation can be drop/dip cast, sprayed onto diverse substrates, like building’s exterior wall (Fig. 4c), glass, metal, wood and plastic. We note that excellent *R̅*_solar_ values (~0.92) can be obtained in regardless of substrate materials (Supplementary Fig. 32 and Supplementary Table 3), which makes our AACP ideal for outdoor roofing material. Specifically, we fabricated half a dozen of AACP coated outdoor wall tiles to demonstrate the cooling performance in a scalable manner (Fig. 4d, Supplementary Figs. 33 and 34, Supplementary Movie 4 and 5). Furthermore, as a proof-of-concept, we measured the inner air temperature of the AACP coated tin box at noon to show its great potential as cool roofing materials (Fig. 4e).

## Discussion

In summary, we demonstrate a strategy for realizing durable radiative cooling coating against harsh environmental aging in the form of paint, namely anti-aging cooling paint (AACP), through evaporation-driven assembled TiO_2_ NPs. Our AACP is fundamentally different from existing radiative cooling materials equipped with hydrophobicity (Supplementary Table 4): the absence of polymer binders in the coatings not only circumvents the UV degrading issue completely, but also leads to much larger difference of the refractive index between TiO_2_ and air compared with the porous polymeric SDRC materials, greatly enhancing the ability of light scattering, thus enabling us to obtain counter-intuitive high *R̅*_solar_ with much thinner coating; the hierarchical morphology formed by evaporation-driven assembled fluorinated TiO_2_ NPs owning a self-similar feature, ensuring the durable self-cleaning function against outdoor aging. The resulting optical and anti-aging performances accompanied by simple fabrication, excellent scalability and cost-effectiveness (Supplementary Fig. 36, Supplementary Table 5) cannot be achieved with existing approaches that involve reflective substrate, multi-layered architectures or complicated post-modification of existing radiative cooling materials, especially when scalability is a desired property. Further exploration of this strategy of implementing inorganic NPs can be extended to a broad range of materials (Supplementary Fig. 35), like calcium carbonate, aluminum oxide, silicon dioxide, barium sulfate and other colored pigments meeting the visual comfort, pushing the SDRC towards the real-world applications.

## Methods

### Raw materials

1H,1H,2H,2H-perfluorooctyltrichlorosilane (PFOTS), rutile type titanium dioxide (TiO_2_) nanoparticles (NPs) (Supplementary Fig. 3), manganese dioxide (MnO_2_) particles, carbon black, ferric oxide (Fe_2_O_3_), humic acid (HAc), sodium chloride (NaCl), sodium nitrate (NaNO_3_), calcium sulfate dihydrate (CaSO_4_ ∙ 2H_2_O), ethanol (95%), acetone (99%), formaldehyde (99%) and toluene (99%) were purchased from Aladdin, Co. All above chemicals were used as received. Deionized (DI) water was produced from Millipore Synergy UV-R water purification system. Borosilicate glass slides (170 µm thick) were purchased from Marienfeld Superior. Adhesives from diverse brands were used obtaining similar performances. Commercial white paint was purchased from Sherwin–Williams (extra white, product number A74W00051). Poly (vinylidene fluoride-co-hexafluoropropylene) (PVDF-HFP, Kynar Flex® 2801) was purchased from Arkema. Silver and aluminum plates, plastic slides (5 mm thick) made of poly(methylmethacrylate) (PMMA), basswood sheet (10 mm thick), soiling agents (including montmorillonite, bentonite, coal ash and sand), aluminum foils, outdoor wall tiles (40 × 80 cm), tin boxes (47 × 32 × 32 cm) and spray coating equipment were purchased from local market.

### Fabrication of anti-aging cooling paint (AACP) and coatings

PFOTS and TiO_2_ NPs were added into ethanol, which was stirred for 5 h at ambient temperature (about 25 °C) to have an apparent uniform suspension. Weight ratio of PFOTS/TiO_2_/ethanol as 0.1/1.3/10 (Supplementary Fig. 12) was routinely used to fabricate AACP suspension. The prepared AACP suspension could be drop/dip-cast or sprayed/brushed onto diverse surfaces to form uniform coatings. In the present work, drop-cast method was chosen to routinely fabricate coatings from AACP. By using scanning electron microscopy to observe the cross-section image of the coating layers, we obtained the coating thickness (Supplementary Fig. 4). The averaged thickness data and corresponding standard deviation were obtained from at least 5 measurements at different positions. To enhance mechanical durability of AACP coatings, a layer of adhesive was first brushed onto the substrate before the drop-cast of AACP, enhancing cohesive strength between the substrate and AACP. Commercial white paint was used as received to brush onto the substrate as control group. Porous coatings from PVDF-HFP were obtained via phase inversion-based process according to previous report^9^. In short, PVDF-HFP and acetone were first mixed to form a solution mixture, then water was added into it to induce liquid-solid phase inversion to have solid coatings. The weight ratio of PVDF-HFP/acetone/water was 1/8/1. For AACP’s scalability demonstration, spray coater was employed for painting on outdoor wall tiles. To obtain uniform coating layer of about 100 µm, a series of equipment parameters were chosen for the purpose, including 0.1 bar as driving pressure, nozzle size of 2.5 mm, rendering 320 mL min^−1^ as flow rate, about 40 cm as spray distance, and spray time of about 1 min to paint the whole tile (40 × 80 cm).

Packing density (*ϕ*) calculation was based on mass conservation principle. The mass density of TiO_2_ NPs is about 4.26 g cm^−3^, measured by Ultrapyc 5000 pycnometer. As an example, for 5 × 5 cm substrate, the used solid content of TiO_2_ suspension is 0.48 g (weight ratio of TiO_2_/ethanol is 1.3/10), the measured dry coating thickness is about 100 µm, then *ϕ* is calculated as $\frac{0.478{{{{{\rm{g}}}}}}/4.26{{{{{\rm{g\; c}}}}}}{{{{{{\rm{m}}}}}}}^{-3}}{25{{{{{\rm{c}}}}}}{{{{{{\rm{m}}}}}}}^{2}\times 100{{{{{\rm{\mu }}}}}}{{{{{\rm{m}}}}}}}\approx 0.45$. In practice, TiO_2_ concentration in ethanol is the determining factor in controlling the magnitude of *ϕ*, high concentration corresponding to high *ϕ* and vice versa.

### Optical characterization of the coatings

The solar reflectance (*R̅*_solar_) of AACP and other coating samples was measured and averaged within wavelength (*λ*) range of 0.3–2.5 µm using PerkinElmer LAMBDA 950 with a polytetrafluoroethylene integrating sphere. The emittance of the coatings was measured within *λ* of 2.5–16 µm using PerkinElmer Spotlight 200I with a gold integrating sphere. The long-wave infrared (LWIR) emittance (${\bar{\varepsilon }}_{{{{{{\rm{LWIR}}}}}}}$) at the transparent atmospheric window was calculated by averaging the emittance data over wavelength of 8–13 µm. For each coating sample, the averaged data and corresponding standard deviation were obtained from at least 3 measurements.

### Surface-wetting characterization

The apparent static water contact angle (*θ*_app_) and roll-off angle (*θ*_roll_) measurements were performed on OCA 50 AF, Dataphysics. For measurement of *θ*_app_, a 6 µL droplet was placed on the coating surface and sat still. The angle between the tangent to the liquid-vapor interface and the solid surface was recorded as *θ*_app_. For measurement of *θ*_roll_, a 10 µL droplet was placed on the coating surface and the substrate was tilted at a speed of 0.1° per second. *θ*_roll_ value was recorded at the moment of droplet-rolling. For each coating sample, the averaged data and corresponding standard deviation were obtained from at least 5 measurements at different positions.

To obtain the energy dissipation factor (*EDF*) of water drops while impacting on the coating surfaces, a 5 µL water droplet was released from a height of 10 mm to impact the coating surface, and the droplet rebounded after impact. The maximum height (*h*, unit of mm) of the droplet rebounding after the first impact was recorded using a high-speed camera (Photron, Fastcam SA5) with 10,000 fps (frames per second). Then the *EDF* is defined as *EDF* = (10 − *h*)/10. For each coating sample, the averaged data and corresponding standard deviation were obtained from at least five measurements at different positions.

### Morphology and other properties characterization

Optical microscopy images were acquired on Nikon LV ND microscope. Scanning electron microscopy (SEM) and energy dispersive X-ray spectroscopy (EDS) analysis were conducted on Phenom Pro X. The morphological and elemental analysis of TiO_2_ NPs were performed by transmission electron microscopy (TEM) using FEI Talos F200S Super-X. Thermogravimetric analysis (TGA) of the coating samples was conducted on Q50 TGA (TA Instrument) with a heating rate of 10 °C min^−1^ under a flow of nitrogen gas (50 mL min^−1^). The functional group analysis was conducted by fourier transform infrared spectroscopy (FTIR, Thermo Fisher Nicolet Is10). The powder X-ray diffraction pattern of TiO_2_ NPs was acquired using X-ray diffractometer (XRD, Bruker D8 Advance). The particle size distribution of TiO_2_ NPs was examined by dynamic light scattering (DLS), Laser particle sizer, Zetasizer Nano ZS-90. Ultra-small angle X-ray scattering (USAXS) experiments were performed on BL10U1 beamline at the Shanghai Synchrotron Radiation Facility. An Eiger-4M detector was used to collect the two-dimensional (2D) scattering data. The x-ray wavelength was 1.24 Å (corresponding energy of 10 keV). The beam size was 400 × 450 µm. All samples were coated on circular mica substrate (almost transparent to x-ray) with 0.1 mm thickness and 30 mm diameter. The distance between the coating and detector was 27,600 mm. The data collection time was 10 s for all the measurements. Fit2D (v12.077) software was used to reduce 2D raw data to one-dimensional (1D) curves. Infrared camera (R300, Mission Technology Co.) was employed to obtain infrared images.

### Finite difference time-domain (FDTD) simulations

FDTD Solutions by Lumerical 2020 R2.4 was employed to investigate the effect of TiO_2_ NPs packing density and nanoparticle size effect on the coating’s solar reflectance. Mesh dimension is 20 × 20 × 20 nm. Due to limited computation power, close-packing model was applied, the film thickness was set as 2 µm and simulated spectrum is 0.25 to 2.5 µm of wavelength. Thickness of 10 and 20 µm were also studied and obtained similar variation trend. Diameter of 200 nm of TiO_2_ NPs was used to generate the date for Fig. 1c. The results are presented in Supplementary Fig. 5.

### Field test for the coatings

All field tests were performed in Chengdu, China. To mimic real operating condition of AACP, we measured the coatings’ temperature variation and cooling power without the implementation of wind and radiation shield. All samples were coated on glass substrates with black tape backing, which could absorb transmitted light, if any. J-type thermocouple was attached on the back of glass and connected to Onset HOBO 4-channel thermocouple data logger UX120-014M. Solar irradiance (*I*_solar_) data were recorded using SP-510 thermopile pyranometer from Apogee Instrument. The thermocouple monitoring ambient temperature was shield by a thermometer shelter to prevent direct solar heating. All samples and instruments were placed on a vacuum insulation panel backing with a styrofoam to limit non-radiative heat transfer. A weather station (DC5V model, YiGu Co.) was used to monitor ambient relative humidity and wind speed (Supplementary Fig. 15). The whole setup was displayed in Supplementary Fig. 17.

The cooling power of the AACP coating was determined by inputting heat power, letting the sample reach ambient temperature. Then, this heat power could be treated as equal to the cooling power of the sample without considering the non-radiative heat transfer. Detailed discussion was in the section of Estimation of radiative cooling power. We used both step-wise and close-tracking method to determine the cooling power respectively. By using step-wise method, we chose to increase heat power in a step-wise manner at noon time, letting the temperature of the sample incrementally reaching steady state within a 5 min time interval. Then we plotted the temperature difference between the sample and ambient as a function of heat power, as shown in Supplementary Fig. 13. The cooling power was then equal to the heat power when the temperature difference reached zero. The heat power was from a customized Kapton® flexible heater controlled by a Keithley 2400 SourceMeter. Proportional-integral-derivative (PID) control via LabVIEW was used to regulate the heat power in response to desired sample temperature value. A thermocouple was attached between the back of the coated glass and the heater to monitor the temperature variation giving feedback data for control accuracy. The sample temperature, heat power and corresponding standard deviation data were obtained over the last 3 min of each time interval after the initial transient peak in heat power. By using close-tracking method, the heater was controlled by the same PID control program to let the AACP surface temperature equal to the ambient temperature in a real-time close tracking manner from 11 AM to 4 PM. Then we can obtain the cooling power variation in a period of 5 h during daytime (Supplementary Fig. 14). All coatings for field tests were on 5 × 5 cm glass substrates.

### Accelerated soiling evaluation based on ASTM D7897-18 standard

We simulated 3 years of natural soiling effect on the coatings according to ASTM D7897-18 standard, which was developed for evaluating soiling effect on the solar reflectance and thermal emittance of roofing materials. According this standard, we prepared the artificial soiling mixture composed of four soiling agents, i.e. soot, dust, particulate organic matter (POM) and salts, as shown in Supplementary Fig. 16. The formulas are as follows:

1. Soot: 0.26 ± 0.01 g of carbon black was mixed with 1 L of distilled water to have a suspension;
2. Dust: A mixture of 0.3 ± 0.02 g of Fe_2_O_3_, 1.0 ± 0.05 g of montmorillonite and 1.0 ± 0.05 g of bentonite was dispensed into 1 L of distilled water to obtain a suspension with a concentration of 2.3 ± 0.1 g/L;
3. POM: 1.4 ± 0.05 g of HAc was mixed with 1 L of distilled water to have a suspension;
4. Salts: A mixture of 0.3 ± 0.03 g of NaCl, 0.3 ± 0.03 g of NaNO_3_ and 0.4 ± 0.03 g of CaSO_4_ ∙ 2H_2_O was dissolved into 1 L of distilled water to obtain a solution with a concentration of 1.0 ± 0.1 g/L.

#### Soiling procedure

The above four soiling agents were mixed to simulate natural soiling materials. The weight ratio of soot/dust/POM/salts was 5/47/28/20. This soiling mixture was poured into a spraying tank, equipped with an air pressure gauge. Then we sprayed the soiling mixture continuously and evenly onto the coatings from a distance of 30 cm above. The sprayed wet soiling agents were controlled as 8 ± 1 mg cm^−2^. The soiled coating was then dried under an infrared heat lamp. Weathering steps before and after soiling procedure were performed to simulate ultraviolet (UV) irradiation and natural aging effect of moisture and rain. This weathering step included 2 cycles of 8 h of UV with 0.89 W m^−2^ at 340 nm and 60 °C, 4 h of simulated rain at 50 °C.

### Additional soiling tests

#### Mud soiling

Mud was formulated by mixing coal ash and water. Coal ash weight ratios of 50% and 80% were produced to simulate low and high viscous mud. We dripped the mud (1 g cm^−2^ for each time) on the coatings to investigate the soiling effect. The soiling process was repeated ten times. The cleaning effect was shown in Supplementary Fig. 21, Supplementary Table 1 and Supplementary Movie 1.

#### MnO_2_ soiling

MnO_2_ particles with diameters ranging from hundreds of nanometers to tens of microns were used to simulate dust contaminations. About 10 mg cm^−2^ of MnO_2_ particles was uniformly spread on the coatings. Water drops and wind-blowing were performed to study cleaning effect respectively. The soiling-cleaning cycle was repeated 10 times. The cleaning effect is presented in Supplementary Fig. 2, Supplementary Table 1 and Supplementary Movie 2.

#### Sand soiling

10 mg cm^−2^ of sand was uniformly spread on the coatings. Water condensation was implemented on the coatings to study the cleaning effect. The coating sample was placed on a cold stage of about 5 °C tilted at 45°. The ambient temperature was about 25 °C and the relative humidity was controlled as 99%. The cleaning effect was shown in Supplementary Fig. 22.

### Accelerated UV irradiation weathering test

We placed the coatings into an UV weathering chamber (HT-UV3, Haotian Testing Equipment Co.). 1000 h (~42 days) of UV irradiance with 0.89 W m^−2^ at 340 nm and 60 °C were used for accelerated weathering experiments. This UV dosage is equivalent to 1 year of Florida sunshine exposure (annual UV dosage of about 275 MJ m^−2^). Florida sunshine exposure is an international benchmark for durability tests of materials.

### Additional stability tests

#### Thermal stability

The AACP coating with adhesives was placed on a heating stage at about 100 °C for 1000 h. The optical and wetting properties after heating were shown in Supplementary Fig. 24.

#### High-speed water jet test

The average jet velocity, *v*, can be calculated as $v=\frac{4V}{\pi {d}^{2}\triangle t}$, where *V* is the volume of water ejected during the time duration of Δ*t* and *d* is the needle diameter. 10 mL of water was jetted out of a 2 mm diameter needle within 400 ms, with an average speed of 8 m s^−1^. This speed is equivalent to the speed of raindrops in the rainstorm (*v* ≈ 9 m s^−1^). Water jet with higher speed out of a faucet (*d* = 5 mm, *v* ≈ 10 m s^−1^) was also used to test the integrity of the AACP coating, as shown in Supplementary Movie 3.

#### Tape-peel test

3 M VHB 5925 tape was applied to test the adhesion force between the coating and substrate. A 1 kg roller was rolled on the tape twice to make the tape hold fast on the coating surface. Then, the tape was peeled from it. The test process and coating properties after tape-peel were shown in Supplementary Fig. 28.

#### Sand falling abrasion test

Sands with diameter of 50 to 300 µm were used to fall on a 24 × 60 mm coating surface from a height of 30 cm. For each time of sand falling, a total sand mass of 20 g was used. The abrasion test was repeated 100 times. The test process and coating properties after sand falling test were shown in Supplementary Fig. 29.

#### Scratch test

Scratch resistance test was performed according to ASTM standard D7027-20 (Test mode A). Specifically, a scratch was applied onto the AACP coating surface under a normal load of 2 N, 20 N and 50 N respectively, over a distance of 0.1 m (±0.0001 m) at a constant scratch rate of 0.1 m s^−1^ (±0.0005 m s^−1^). The coating properties after scratch test are shown in Supplementary Fig. 30 and Supplementary Table 4.

#### Photocatalysis effect of AACP coating

The coating sample was on glass substrates (22 × 60 mm) with a thickness of about 100 μm. The concentrations of the feeding stream of formaldehyde and toluene were both controlled as 30 ppm in nitrogen gas flow. The gas humidity was controlled at 50%, and the total flow rate was set as 25 mL/min. A UV lamp (300 W) was vertically placed above the reactor (0.357 L). The incident light wavelength was controlled at 365 nm and the light intensity 0.29 W cm^−2^. The lamp was turned on after reaching the adsorption-desorption equilibrium. The concentrations of formaldehyde, toluene, water and CO_2_ were continuously recorded by a multi-gas analyzer (DKG-42A, Duke Technology). The pollutant conversion efficiency was calculated as *η* (%) = (C_0_ − C)/C_0_ × 100%, where *C*_*0*_ and *C* representing the concentration of formaldehyde or toluene in the feeding stream and the real-time concentration in the outlet stream, respectively^42^. The results are shown in Supplementary Fig. 31.

### Outdoor long-term durability test at real-world condition

Two locations in China were selected to demonstrate AACP’s outdoor long-term durability performance. One was Chengdu (30°40'36''N, 104°6'28''E) in humid and hot climate, dating from April 25th to November 10th, 2021. The other one was Xi’an (34°17'57''N, 108°58'21''E) in dry and hot climate, from May 1st to November 30th, 2021. The coating samples (5 × 15 cm) were fixed on a display board (80 × 120 cm) tilted at 30° facing the sky. All coating slides were split into three regions: unexposed/(exposed, washed)/(exposed, unwashed). We used aluminum foil to wrap one third of the slide as “unexposed” region. The “wash” was done by tap water rinsing. When the outdoor exposure was finalized, the samples were retrieved to test the optical and wetting properties. The results of aging of the samples in Xi’an were presented in Supplementary Fig. 25.

### Definitions of solar reflectance and thermal emittance

The solar reflectance (*R̅*_solar_) is defined as the ratio of the reflected solar power in the *λ* of 0.3–2.5 µm to the integral of solar intensity within the same wavelength range^43^:

1 $${\bar{R}}_{{{{{{\rm{solar}}}}}}}=\frac{{\int }_{0.3\,{{{{{\rm{\mu }}}}}}{{{{{\rm{m}}}}}}}^{2.5\,{{{{{\rm{\mu }}}}}}{{{{{\rm{m}}}}}}}{I}_{{{{{{\rm{solar}}}}}}}\left(\lambda \right)R\left(\lambda \right){{{{{\rm{d}}}}}}\lambda }{{\int }_{0.3\,{{{{{\rm{\mu }}}}}}{{{{{\rm{m}}}}}}}^{2.5\,{{{{{\rm{\mu }}}}}}{{{{{\rm{m}}}}}}}{I}_{{{{{{\rm{solar}}}}}}}\left(\lambda \right){{{{{\rm{d}}}}}}\lambda },$$

where *I*_solar_(*λ*) is the ASTM G173-03 AM 1.5 Global Tilt spectrum, *R*(*λ*) is the measured spectral reflectance of the sample.

In a similar way, the thermal emittance (${\bar{\varepsilon }}_{{{{{{\rm{LWIR}}}}}}}$) is defined as the ratio of the sample’s thermal radiation energy in the primary long-wave infrared (LWIR) atmospheric transparency window (*λ* of 8–13 µm) to the integral of spectral intensity emitted by a standard blackbody within the same wavelength range:

2 $${\bar{\varepsilon }}_{{{{{{\rm{LWIR}}}}}}}=\frac{{\int }_{8\,{{{{{\rm{\mu }}}}}}{{{{{\rm{m}}}}}}}^{13\,{{{{{\rm{\mu }}}}}}{{{{{\rm{m}}}}}}}{I}_{{{{{{\rm{bb}}}}}}}\left(T,\,\lambda \right)\varepsilon \left(T,\,\lambda \right){{{{{\rm{d}}}}}}\lambda }{{\int }_{8\,{{{{{\rm{\mu }}}}}}{{{{{\rm{m}}}}}}}^{13\,{{{{{\rm{\mu }}}}}}{{{{{\rm{m}}}}}}}{I}_{{{{{{\rm{bb}}}}}}}\left(T,\,\lambda \right){{{{{\rm{d}}}}}}\lambda },$$

where $\varepsilon \left(T,\lambda \right)$ is the sample’s measured spectral emittance and *I*_bb_(*T*, *λ*) is the blackbody radiation intensity at a temperature of *T* calculated by Planck’s law:

3 $${I}_{{{{{{\rm{bb}}}}}}}\left(T,\lambda \right)=\frac{2{c}^{2}{{\hbar }}}{{\lambda }^{5}}\frac{1}{{e}^{\frac{{{\hbar }}c}{\lambda {k}_{{{{{{\rm{B}}}}}}}T}}-1},$$

where $\hslash$ is the Planck constant, *c* is the speed of light in a vacuum, *k*_B_ is the Boltzmann constant. In the present work, the temperature *T* is set as 298 K.

### Estimation of radiative cooling power

To evaluate the cooling performance of the radiative cooling materials, the subambient temperature drop is a parameter to feel the cooling ability intuitively. However, since the test conditions, like location, wind speed and humidity, etc. could vary case by case, one can hardly obtain the same temperature reduction even for the same material from different tests^44^. Therefore, it is more appropriate to use cooling power for objectively comparing the cooling ability of various materials.

To estimate the cooling power of a radiative cooling surface, we start from the steady state heat transfer balance analysis^45^. For a radiative cooling surface with temperature $T$ and ambient temperature *T*_a_, the net cooling power *P*_net_(*T*, *T*_a_) is calculated as

4 $${P}_{{{{{{\rm{net}}}}}}}\left(T,{T}_{{{{{{\rm{a}}}}}}}\right)={P}_{{{{{{\rm{rad}}}}}}}\left(T\right)-{P}_{{{{{{\rm{atm}}}}}}}\left({T}_{{{{{{\rm{a}}}}}}}\right)-(1-{\bar{R}}_{{{{{{\rm{solar}}}}}}}){P}_{{{{{{\rm{sun}}}}}}}-{P}_{{{{{{\rm{nrad}}}}}}}\left(T,{T}_{{{{{{\rm{a}}}}}}}\right),$$

where *P*_rad_(*T*) is emitted power from the surface, *P*_atm_(*T*) is absorbed power from atmospheric radiation, *P*_sun_ is energy power from the sun and *P*_nrad_(*T*, *T*_a_) is the non-radiative heat transfer power stemming from the surrounding environmental conductive and/or convective heat exchange. For a radiative cooling surface with a unit area of 1 m^2^, *P*_nrad_(*T*, *T*_a_) can be further expressed as *h*_c_(*T*_a_ – *T*) by bringing in a non-radiative heat transfer coefficient *h*_c_ (unit of W m^−2^ K^−1^). Like we mentioned before, real environmental conditions could strongly influence the *h*_c_ making it vary from 2 to 20 W m^−2^ K^−1^ ^44^. Hence, to rule out this uncontrolled factor, we can set Δ*T* = *T*_a_ – *T* *=* 0, thus eliminating the *P*_nrad_(*T*, *T*_a_) term. In practice, we utilized a heater to compensate the heat loss of the cooling device to make its temperature equal to the ambient one. Then, the net cooling power *P*_net_(*T*, *T*_a_) becomes *P*_net_(*T*_a_, *T*_a_) and can be further defined as *P*_cool_(*T*_a_), which equals to the heat power.

Now let us consider an ideal scenario, i.e. the radiative cooling material has 100% reflectance in the solar spectrum (*λ* of 0.3–2.5 µm) and 100% emittance in the primary atmospheric transparency window (*λ* of 8–13 µm). Then, (1 − *R̅*_solar_)*P*_sun_ = 0.

5 $${P}_{{{{{{\rm{rad}}}}}}}\left({T}_{{{{{{\rm{a}}}}}}}\right)=2\pi {\int }_{0}^{\frac{\pi }{2}}{{{{{\rm{cos }}}}}}\theta {{{{{\rm{sin }}}}}}\theta {\int }_{0}^{{{\infty }}}{I}_{{{{{{\rm{bb}}}}}}}\left({T}_{{{{{{\rm{a}}}}}}},\,\lambda \right)\varepsilon \left(\lambda,\,\theta \right){{{{{\rm{d}}}}}}\lambda {{{{{\rm{d}}}}}}\theta,$$

6 $${P}_{{{{{{\rm{atm}}}}}}}\left({T}_{{{{{{\rm{a}}}}}}}\right)=2\pi {\int }_{0}^{\frac{\pi }{2}}{{{{{\rm{cos }}}}}}\theta \,{{{{{\rm{sin }}}}}}\theta {\int }_{0}^{{{\infty }}}{I}_{{{{{{\rm{bb}}}}}}}\left({T}_{{{{{{\rm{a}}}}}}},\,\lambda \right)\varepsilon \left(\lambda,\,\theta \right){\varepsilon }_{{{{{{\rm{a}}}}}}}\left(\lambda,\,\theta \right){{{{{\rm{d}}}}}}\lambda {{{{{\rm{d}}}}}}\theta,$$

and

7 $${\varepsilon }_{{{{{{\rm{a}}}}}}}\left(\lambda,\theta \right)=1-{{t}_{{{{{{\rm{a}}}}}}}\left(\lambda,0\right)}^{1/{{{{{\rm{cos }}}}}}\theta },$$

where $\varepsilon (\lambda,\theta )$ and ${\varepsilon }_{{{{{{\rm{a}}}}}}}\left(\lambda,\theta \right)$ are spectral and angular emissivity of the radiative cooling surface and ambient air, *t*_a_(*λ*, 0) is the atmospheric transmittance at the zero zenith angle. In our estimation, the data of *t*_a_(*λ*, 0) is from ATRAN modeling software. The cooling power mainly relies on the surface spectral emittance through the primary atmospheric transparency window with *λ* of 8 to 13 µm. Then we obtain *P*_cool_(*T*_a_) as

8 $${P}_{{{{{{\rm{cool}}}}}}}\left({T}_{{{{{{\rm{a}}}}}}}\right)=2\pi {\int }_{0}^{\frac{\pi }{2}}{{{{{\rm{cos }}}}}}\theta {{{{{\rm{sin }}}}}}\theta {\int }_{8\,{{{{{\rm{\mu }}}}}}{{{{{\rm{m}}}}}}}^{13\,{{{{{\rm{\mu }}}}}}{{{{{\rm{m}}}}}}}{I}_{{{{{{\rm{bb}}}}}}}\left({T}_{{{{{{\rm{a}}}}}}},\,\lambda \right)\varepsilon \left(\lambda,\,\theta \right){{t}_{{{{{{\rm{a}}}}}}}\left(\lambda,\,0\right)}^{1/{{{{{\rm{cos }}}}}}\theta }{{{{{\rm{d}}}}}}\lambda {{{{{\rm{d}}}}}}\theta .$$

As a result, *P*_cool_ as a function of ambient temperature *T*_a_ can be plotted as shown in Fig. 1a of the main text. It is worth noting that we set *R̅*_solar_ = 1 to eliminate the absorbed solar power in the above discussion. We can study the impact of imperfect *R̅*_solar_ simply by reintroducing non-zero term of (1 − *R̅*_solar_)*P*_sun_ to have the net cooling energy *P*_net_(*T*_a_):

9 $${P}_{{{{{{\rm{net}}}}}}}\left({T}_{{{{{{\rm{a}}}}}}}\right)={P}_{{{{{{\rm{cool}}}}}}}\left({T}_{{{{{{\rm{a}}}}}}}\right)-(1-{\bar{R}}_{{{{{{\rm{solar}}}}}}}){P}_{{{{{{\rm{sun}}}}}}}.$$

### Energy saving analysis on a medium size building level

We estimate the saved electricity on a three-storey commercial office building with a 1600 m^2^ roof for AACP and reflective white paint for a 3-year period. The simulation procedure is similar to the previous work.^5^ EnergyPlus (version 22.1.0) was utilized to determine the electricity usage with a standard heating, ventilation and air conditioning (HVAC) system based on hourly weather data of Singapore city (hot climate) acquired from the International Weather for Energy Calculation (IWEC) files. The interior temperature of the building was set as 24 °C at all time and HVAC system operating continuously. The cooling power of AACP was calculated according to Eq. (9) with input of ambient temperature, solar irradiance and cloud coverage data, then subtracted from the heat load for the building. Coefficient of performance (COP) of 2.8 was used to calculate the remaining heat dissipated in the building. The reflectance and emittance of the roof of the default reference building were fixed at 0.3 and 0.9. For white painted roof, the emittance was fixed at 0.9. The reflectance was set with a declining trend as 0.87, 0.82, and 0.78 for each year in a period of 3 years. For AACP, the reflectance was set as 0.93, 0.928, and 0.926 for each year, and emittance was fixed at 0.97. The simulation results are presented in Supplementary Fig. 36. Due to environmental aging, the electricity saving for white paint decreases each year from 12.2 to 8.4 kWh m^−2^, while AACP renders robust energy saving performance above 50 kWh m^−2^. In Supplementary Table 5, we compared the cost between AACP and two other common white paint based on one square meter. We find that the cost of AACP is comparable to the prices of the commercial products (~$5 m^−2^ *vs*. $4 m^−2^). In this case, the electricity saving difference from AACP than white paint is ~40 kWh m^−2^ for 1 year. We assume the grid electricity cost is $0.1 kWh^−1^, then the cost difference between AACP and white paint can be compensated in the first year or maybe in even shorter period, which shows the great potential for AACP to be a cost effective solution as cool roof to mitigate energy demand for heavy cooling load.

## Supplementary information

Supporting Information

Description of Additional Supplementary Files

Supplementary Movie 1

Supplementary Movie 2

Supplementary Movie 3

Supplementary Movie 4

Supplementary Movie 5

## Data Availability

The data that support the findings of this study are available from the corresponding authors upon reasonable request.
