# Supplementary material for: Durable radiative cooling against environmental aging
Source: Nat Commun. 2022 Aug 16;13:4805. doi: 10.1038/s41467-022-32409-7 (PMC9381728; doi:10.1038/s41467-022-32409-7)
Supplement: Supplementary file 1 — Supporting Information [file 41467_2022_32409_MOESM1_ESM.pdf]

Supplementary Materials for

Durable radiative cooling against environmental aging

Jianing Song†, Wenluan Zhang†*, Zhengnan Sun, Mengyao Pan, Feng Tian, Xiuhong Li, Ming Ye, Xu Deng*

*Correspondence to: wenluanzh@uestc.edu.cn (W.Z.); dengxu@uestc.edu.cn (X.D.)
†These authors contributed equally to this work.

This PDF file includes:

Supplementary note
Figs. 1 to 36
Tables 1 to 5
References

Other Supplementary Materials for this manuscript include the following:

Supplementary Movies 1 to 5 (.avi)

Supplementary note

<u>Scattering efficiency of a single sphere based on Mie theory</u>

Mie theory offers a precise solution to the scattering of plane electromagnetic waves from a homogenous sphere calculated directly from Maxwell equations. It gives us qualitative understanding of the relationship between the scattering efficiency of a single NP and the reflectance of resulted coatings. The scattering cross-section (C_{sca}) of a spherical particle is derived as(*1*)

$$C_{\mathrm{sca}} = \frac{2\pi}{k^2}\sum_{n=1}^{\infty}(2n+1)\left(|a_n|^2+|b_n|^2\right),\#(1)$$

$$a_n = \frac{m\psi_n(mx)\psi_n'(x)-\psi_n(x)\psi_n'(mx)}{m\psi_n(mx)\xi_n'(x)-\xi_n(x)\psi_n'(mx)},\#(2)$$

$$b_n = \frac{\psi_n(mx)\psi_n'(x)-m\psi_n(x)\psi_n'(mx)}{\psi_n(mx)\xi_n'(x)-m\xi_n(x)\psi_n'(mx)},\#(3)$$

where k is wavevector, n is multipole order, a_n is n^{th} electric mode coefficient, b_n is n^{th} magnetic mode coefficient, m is the relative refractive index, ψ and ξ are Riccati-Bessel functions. Then the scattering efficiency coefficient (Q_{sca}) is the normalization of C_{sca} as

$$Q_{\mathrm{sca}} = \frac{C_{\mathrm{sca}}}{\pi r^2},\#(4)$$

where πr^2 is the geometrical cross-sectional area of the scattering particle. In the present work, we used MiePlot program (v4.6.19), developed by Philip Laven, to obtain the scattering efficiency from a single NP as a function of wavelength and NP diameter(*2*). The refractive index of TiO_2 was extracted from previous works(*3, 4*).

<u>Surface adhesion force analysis</u>

The surface adhesion force F_{adh} between a flat surface and a spherical particle is given by the JKR (Johnson-Kendall-Roberts) theory(*5*):

$$F_{\mathrm{adh}} = \frac{3}{4}\pi D(\gamma_{\mathrm{surf}}+\gamma_{\mathrm{part}}-\gamma_{\mathrm{sp}}) \quad (5)$$

where D is the particle diameter, γ_{surf} is the surface energy of the substrate, γ_{part} is the surface energy of the particle. γ_{sp} is the interfacial energy between the substrate and particle, which can be neglected in this case. For superhydrophobic (SH) surface, we insert φ as the ratio of the actual

to apparent contact area of the particle to obtain the adhesion force of SH surface, $F_{\mathrm{adh}}^{\mathrm{SH}}$. Therefore, the Equation S13 becomes $F_{\mathrm{adh}}^{\mathrm{SH}} = \varphi F_{\mathrm{adh}}$. We estimated the γ_{surf} of the hydrophobic surface (fluorinated flat surface) as 10 mN m^{-1} and γ_{surf} of the hydrophilic surface as 100 mN m^{-1}, φ as 0.1(*6, 7*). Then we plot the F_{adh} as a function of D. Fig. 11 exhibits that the adhesion force of both hydrophilic and hydrophobic particles on the SH surface is much lower than that on other surfaces, which is also the main reason why the dust particles on our AACP coatings are easy to be washed and blown off.

Ultra-small X-ray scattering data analysis

In Fig. 8, USAXS data are first presented in the form of log-log profile. The scattering curves cover wave-vector (q) from 6×10^{-4} to 2.7×10^{-2} Å^{-1}, corresponding to d-spacing from approximately 23 nm to 1 μm. Since TiO_2 NPs form continuous phase as the solid content of the coatings, we treat formed air pores within the coatings as solute scatters, TiO_2 matrix as solvent. Although the obtained scattering intensity was not calibrated absolute intensity, since the coating thickness of all samples are about the same as 100 μm, we can still roughly compare the scattering intensity of the samples. In general, for the same form factor, $\mathrm{I}(q) \propto \Delta\rho^2 \cdot V$, where $\Delta\rho$ is scattering length density contrast, V is the volume fraction (vol%) of the scatter. In our case, $\Delta\rho$ origins from the contrast between air pore and TiO_2 NPs, which is unchanged through the course. Hence, the decline of I(q) at low-q regime is consistent with the decrease of the air pore vol%(*8*).

By plotting $\mathrm{I}(q)\cdot q^2$ *vs.* q, we obtained Kratky plot allowing further investigation of the coating's bulk morphology. The peak in the low-q regime features particle-like nature, which indicates the presence of air pores. For sample with 0.33 vol% of TiO_2 NPs, considering $\mathrm{I}(q)\cdot q^2 \rightarrow 0$, when $q \rightarrow 0$. Hence, the upturn of the curve makes this curve must form a peak in the ultra-low q regime and finally reaches the origin of the axis, which is beyond the detectable low q value of our facility. In this way, this upturn of the curve is suggestive of a structure featured with micrometer length scale. For the other samples, there is a strong peak (peak S) at about 1.8×10^{-3} Å^{-1} (d-spacing of about 350 nm, big air pore), and a weak shoulder (shoulder W) at about 5.7×10^{-3} Å^{-1} (d-spacing of about 110 nm, small air pore). With the denser packing of TiO_2 NPs (meaning less air pores), peak S diminishes and shoulder W becomes more significant. This variation suggests, with dense TiO_2 NPs packing, relative to small air pore, the volume fraction of big air pore decreases more significantly. Overall, USAXS data demonstrate this coating having bulk morphology of

hierarchical structure featured with air pores at two different length scales. A clear hierarchical structure evolution is observed with the dense packing of TiO_2 NPs.

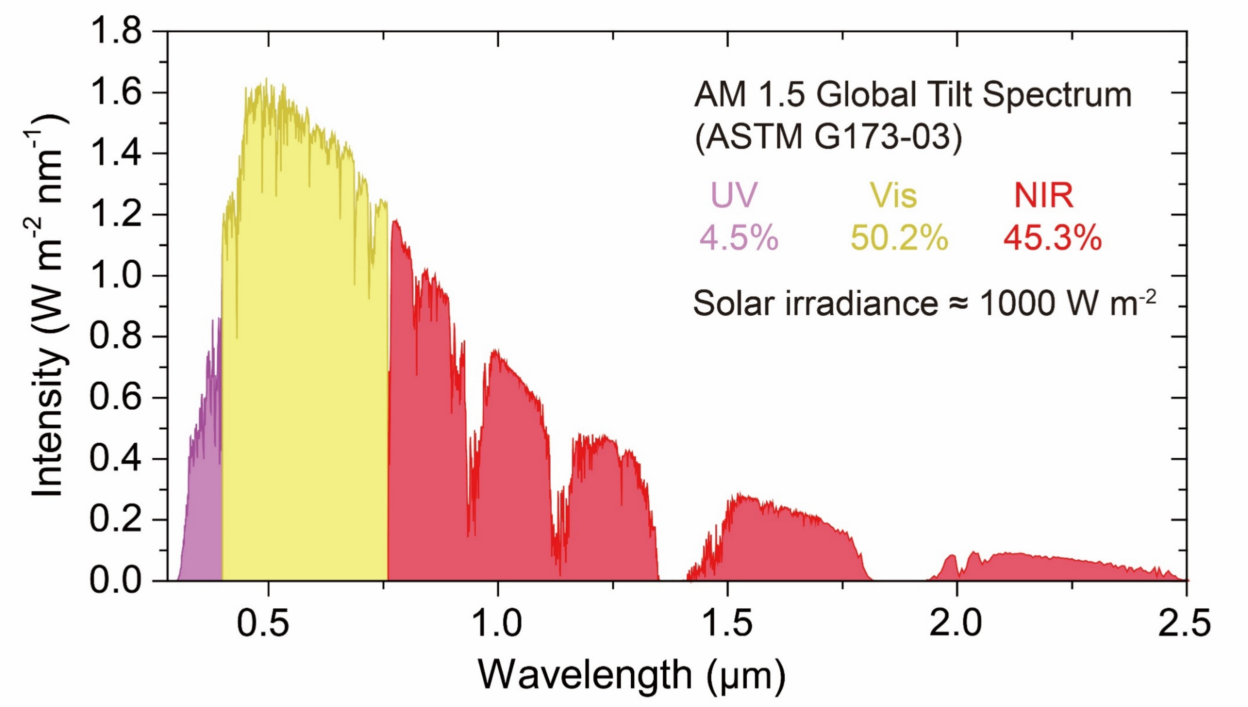

Supplementary Fig. 1. AM 1.5 standard solar spectrum based on ASTM G173-03. The energy proportion within UV region (wavelength of 0.28 to 0.4 μm) only accounts for 4.5% of the whole spectrum. The visible (Vis) and near infrared (NIR) regions account for 50.2% and 45.3% of the whole spectrum, respectively. Therefore, the use of TiO_2 NPs does not affect the $\overline{R}_{solar}$ in a great deal in the sense of whole solar spectrum.

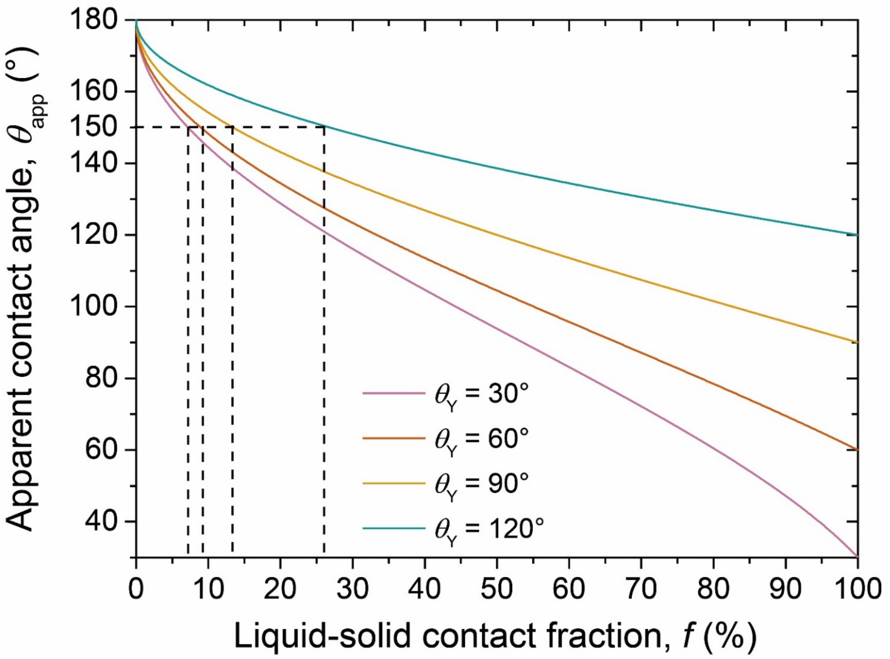

Supplementary Fig. 2. The Cassie-Baxter equation derived plots. Apparent contact angle (θ_{app}) as a function of liquid-solid contact fraction (f) and Young's contact angle (θ_Y). For flat smooth surface, the largest θ_Y is approximately 120°.

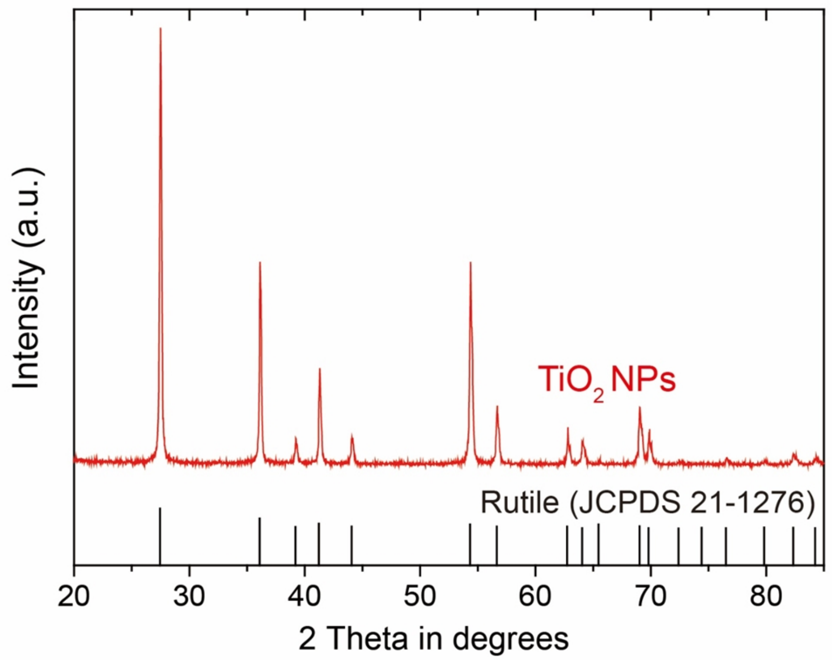

Supplementary Fig. 3. Powder XRD pattern of TiO_2 NPs showing rutile type structure.

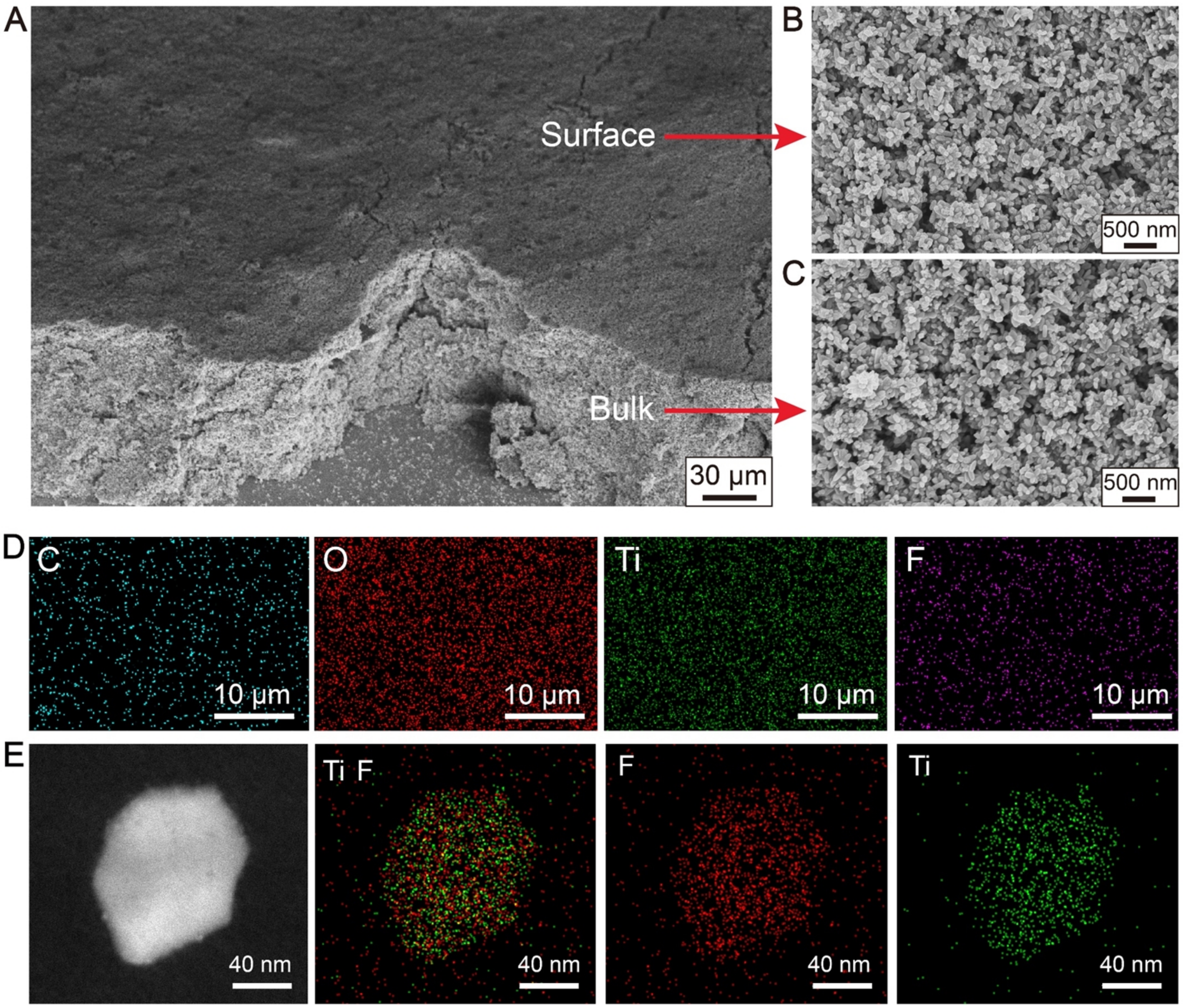

Supplementary Fig. 4. SEM and TEM characterizations of AACP. (**A**) SEM image showing full view of the AACP coating. (**B**) Top and (**C**) side views showing similar morphologies in SEM images. (**D**) EDS mapping of AACP coating showing the existence of carbon (C), oxygen (O), titanium (Ti) and fluorine (F). (**E**) Dark-field TEM image and EDS mapping of single fluorinate TiO_2 nanoparticle showing the existence of titanium (Ti) and fluorine (F).

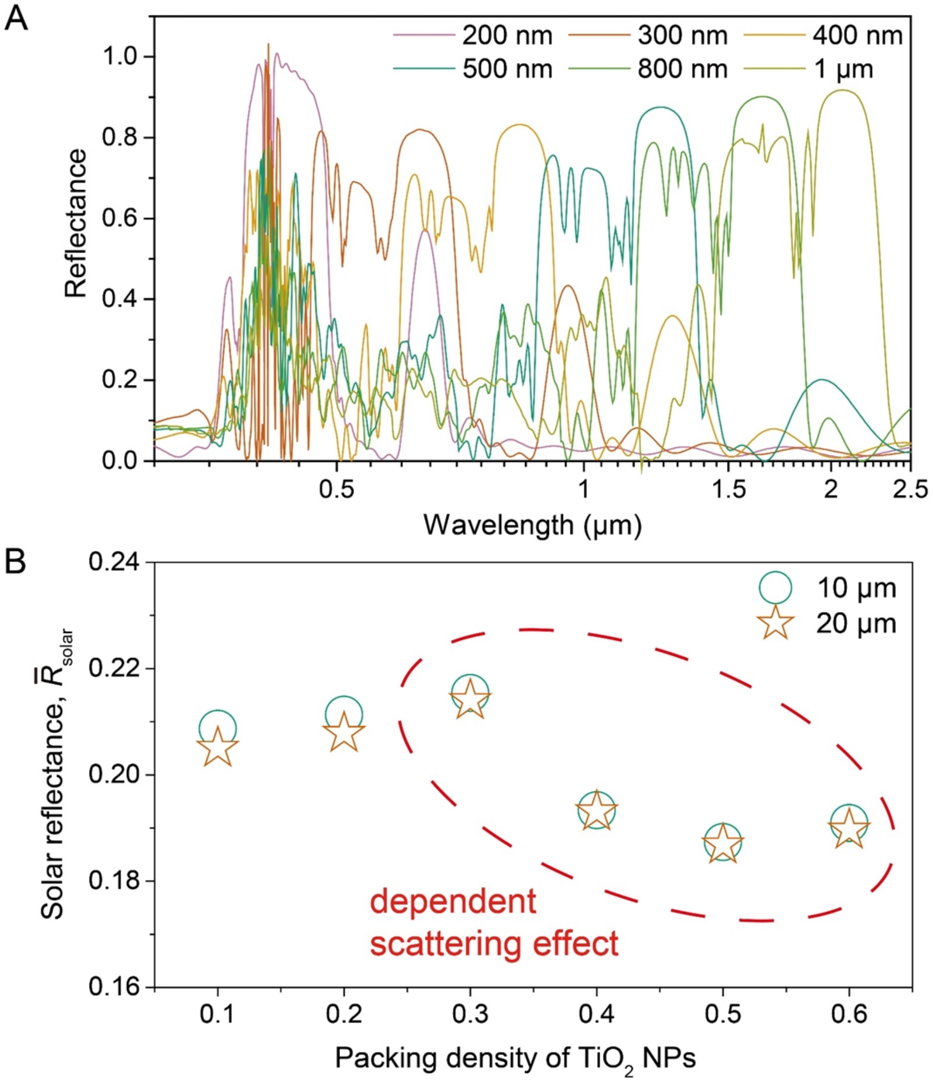

Supplementary Fig. 5. FDTD simulation results. (**A**) TiO_2 NPs with various diameters can collectively cover the whole solar spectrum. Packing density of 0.3 and 2 μm of thickness were used for all simulations above. (B) Coatings with thicknesses of 10 and 20 μm have similar declining trend of reflectance as shown in the main text Fig. 1c, due to the dependent scattering effect.

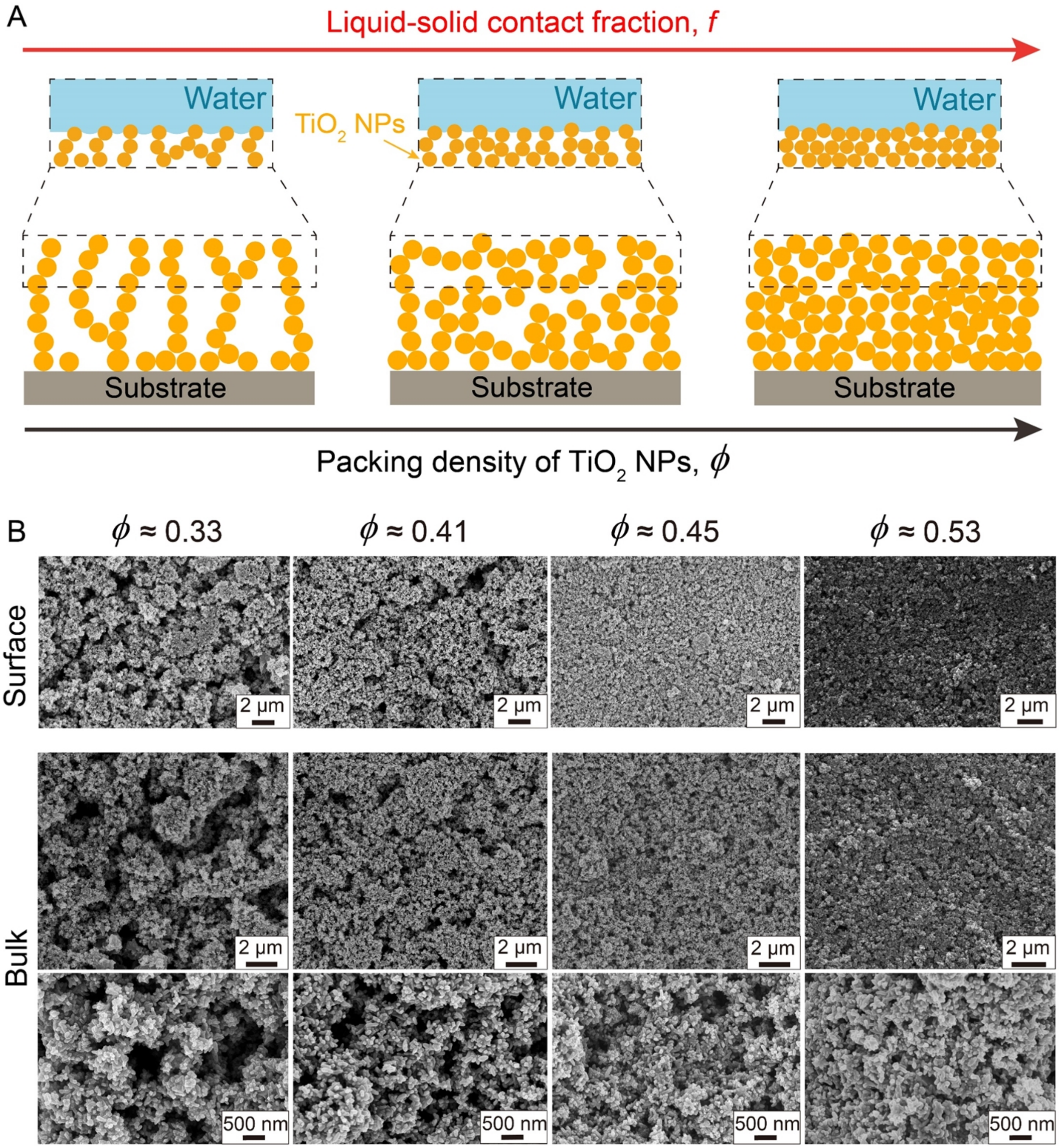

Supplementary Fig. 6. The surface and bulk of the coating showing similar morphologies. (**A**) Schematic illustrating the packing density (ϕ) affecting the surface and bulk morphology in the same trend. (**B**) SEM top and side-view images showing the packing density (ϕ) effect on the AACP's coating's surface and bulk, which show similar morphology. Hence, the liquid-solid contact fraction, f, should be in direct proportion to the magnitude of ϕ.

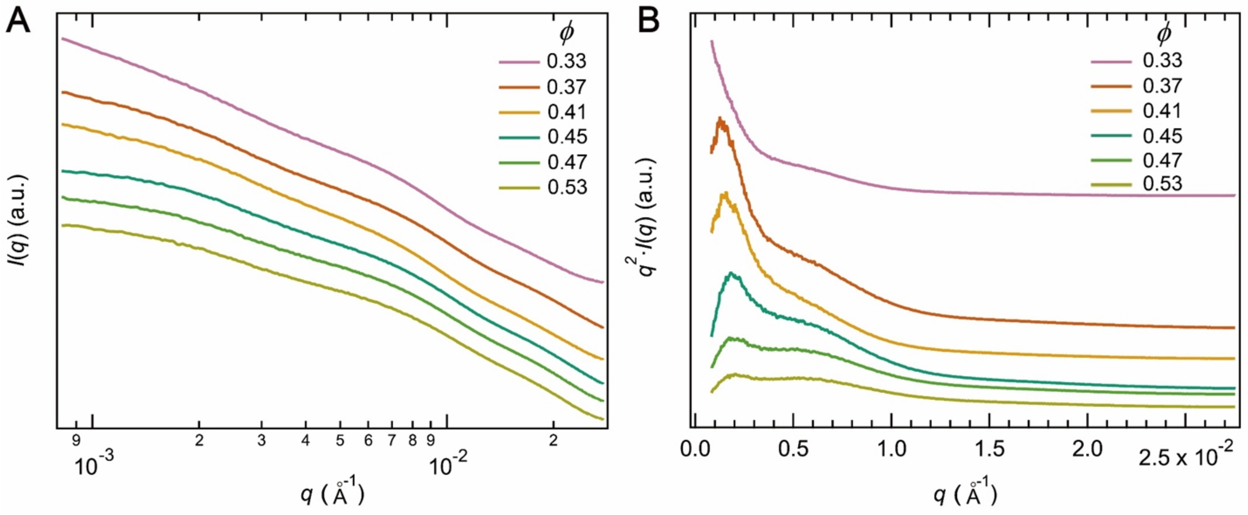

Supplementary Fig. 7. USAXS characterizations. USAXS data in (**A**) log-log profile and (**B**) Kratky plot showing the hierarchical porous nature of the bulk of AACP coating.

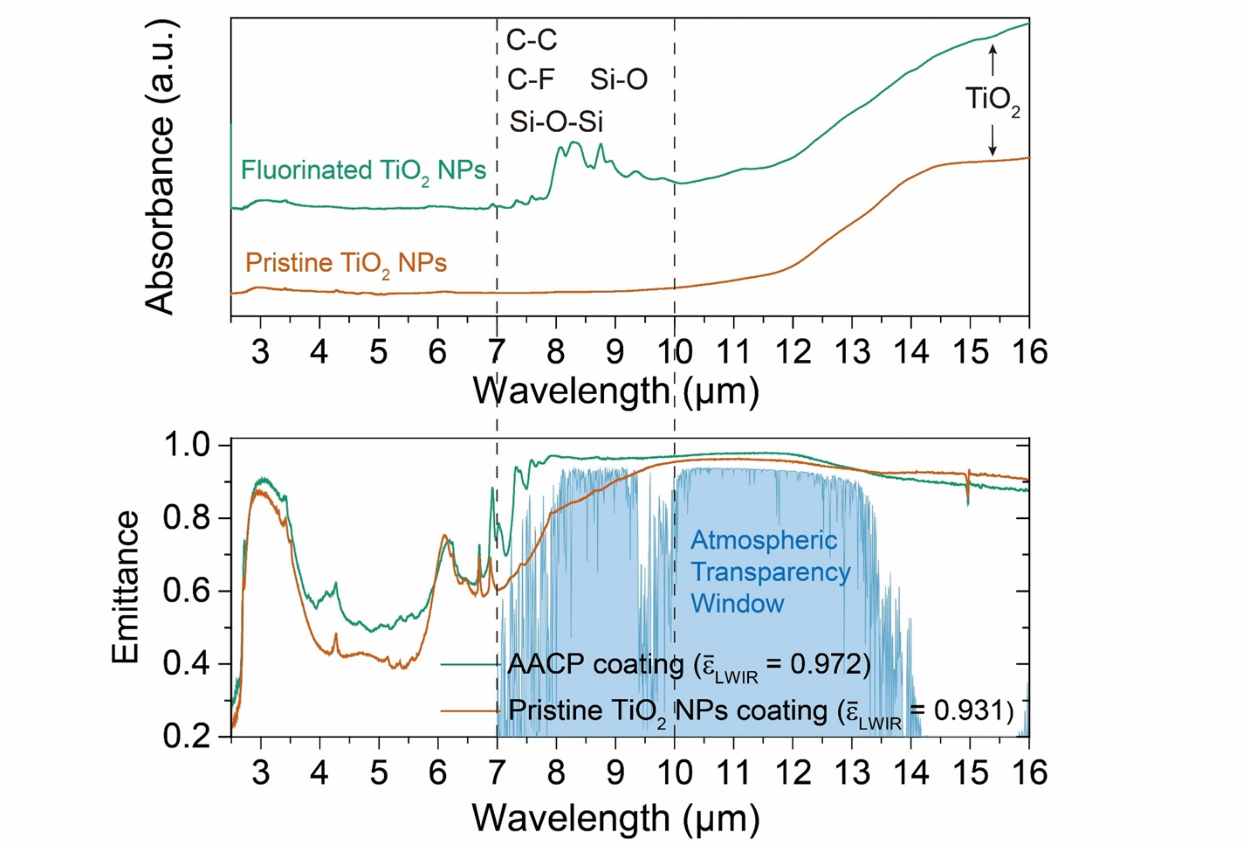

Supplementary Fig. 8. Fourier-transform infrared spectroscopy and emittance data. The strong coupling of the C−F, C−C, Si−O and Si−O−Si stretching vibrations leads to the emittance enhancement within the transparent atmospheric window (8 to 13 μm of wavelength).

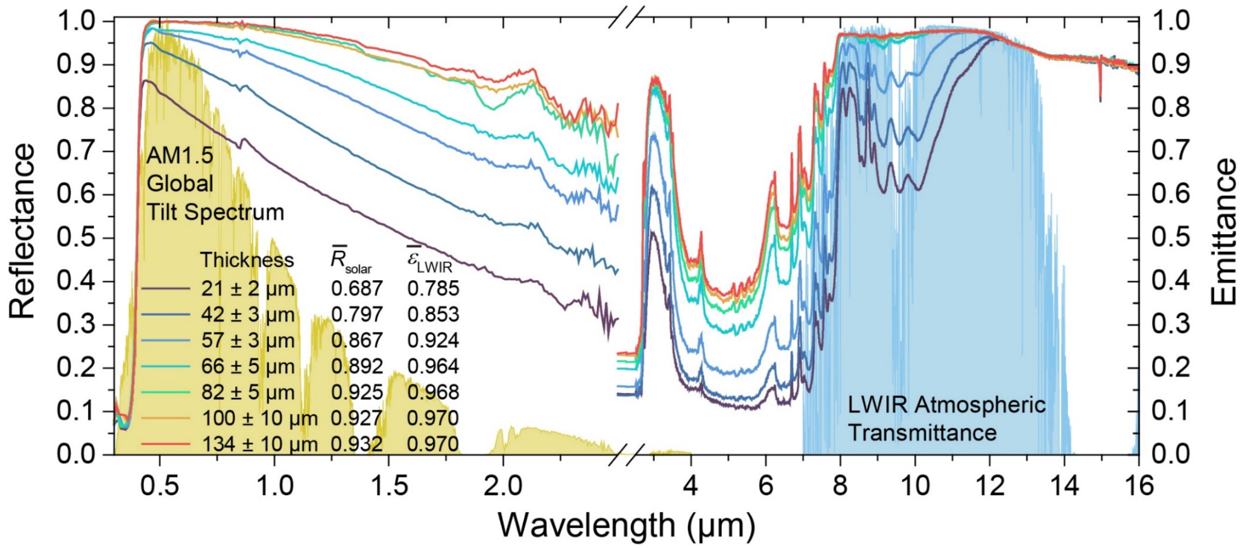

Supplementary Fig. 9. Coating thickness effect on $\boldsymbol{\overline{R}_{solar}}$ and $\boldsymbol{\bar{\varepsilon}_{LWIR}}$. As the coating thickness increases, the $\overline{R}_{solar}$ experiences a rise until the thickness of 82 µm. The $\boldsymbol{\bar{\varepsilon}_{LWIR}}$ does not change much when the thickness reaches 66 µm. In the present work, we controlled the thickness as 100 µm as a routine. We carefully selected substrates with optical properties, i.e. $\overline{R}_{solar}$ and $\boldsymbol{\bar{\varepsilon}_{LWIR}}$, having little effect on the coatings' measured values. Specifically, we used borosilicate glass ($\overline{R}_{solar} \approx 0.09$) for reflectance measurements, and silver plate ($\boldsymbol{\bar{\varepsilon}_{LWIR}} \approx 0.1$) for emittance measurements.

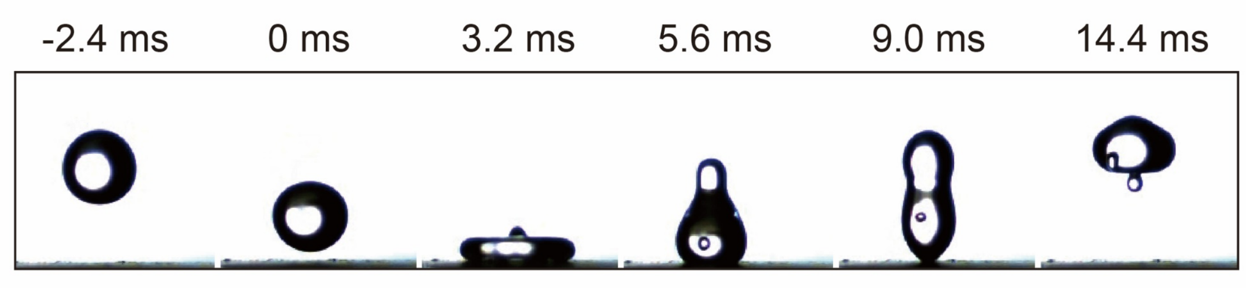

Supplementary Fig. 10. Time-resolved images of the bouncing of a 5 µL water droplet on the AACP coating surface with nanoparticles packing density (ϕ) of 0.45.

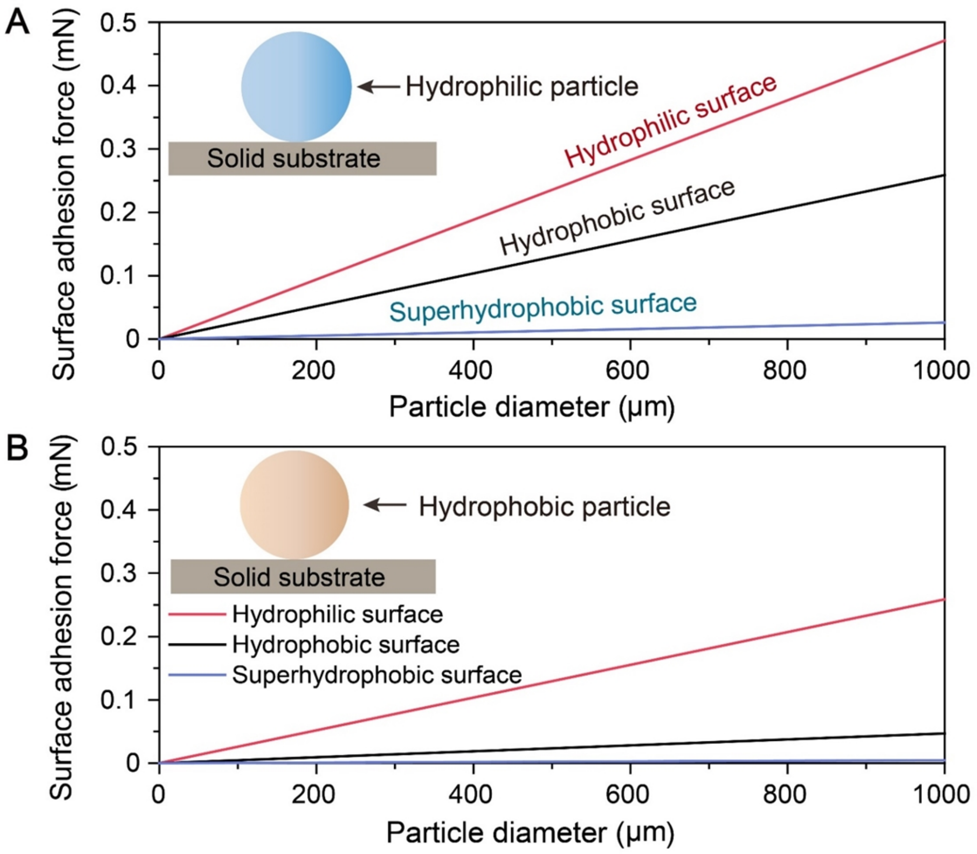

Supplementary Fig. 11. The surface adhesion force of coatings. (**A**) Hydrophilic (γ_{part} = 100 mN m^{-1}) and (**B**) hydrophobic particle (γ_{part} = 10 mN m^{-1}) on surfaces as functions of coating surface energy and particle diameter. Our AACP coating has a superhydrophobic nature, thus featuring the lowest surface adhesion force, beneficial to anti-soiling function.

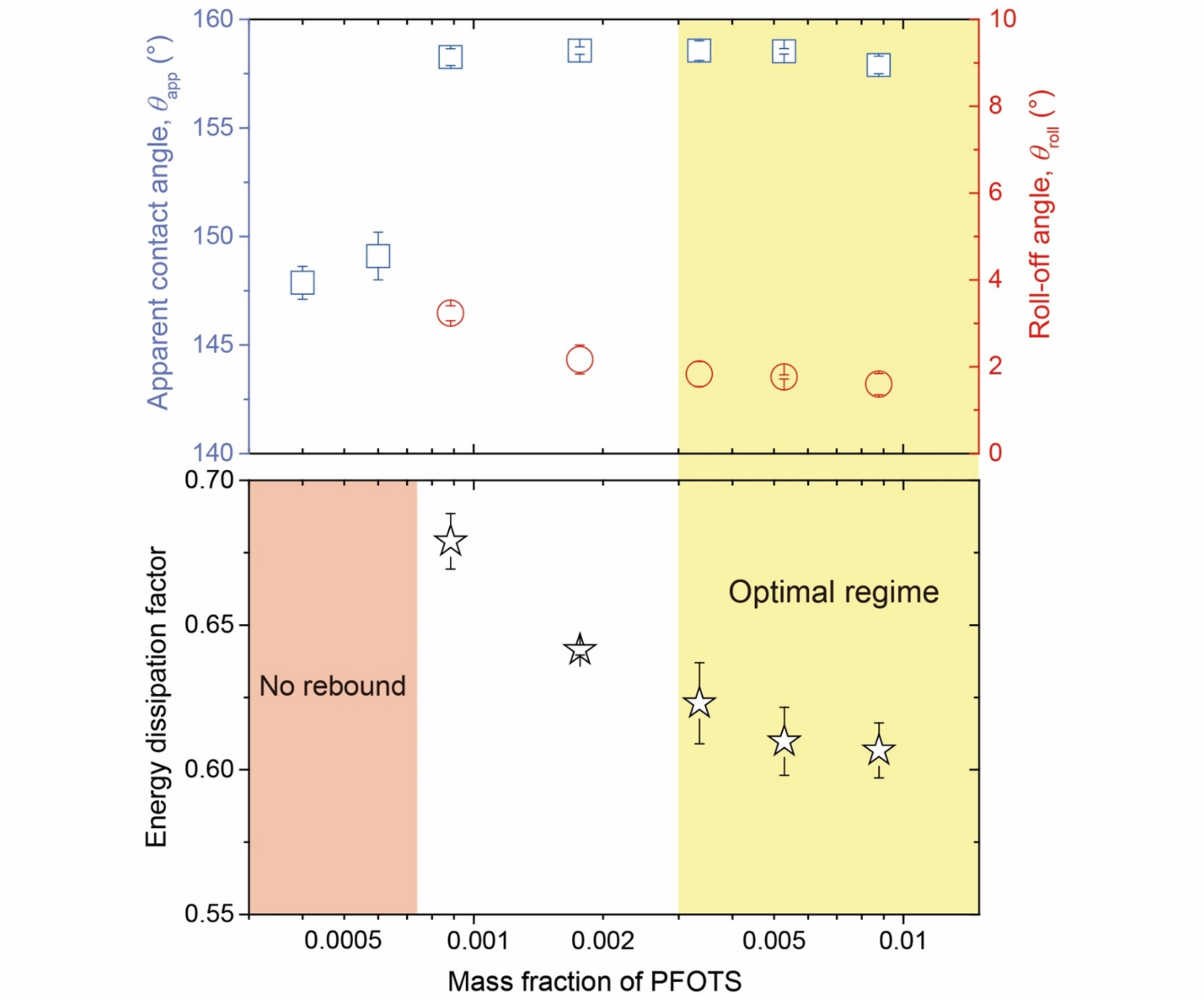

Supplementary Fig. 12. θ_{app}, θ_{roll} and energy dissipation factor (*EDF*) of AACP coating as functions of mass fraction of PFOTS. To obtain super-repellent surfaces with optimal non-wetting properties, the mass fraction of PFOTS should be larger than 0.003. In practice, we used a weight ratio of 0.1/1.3/10 (PFOTS mass fraction of 0.009) for PFOTS/TiO_2/ethanol as a routine to fabricate AACP coatings.

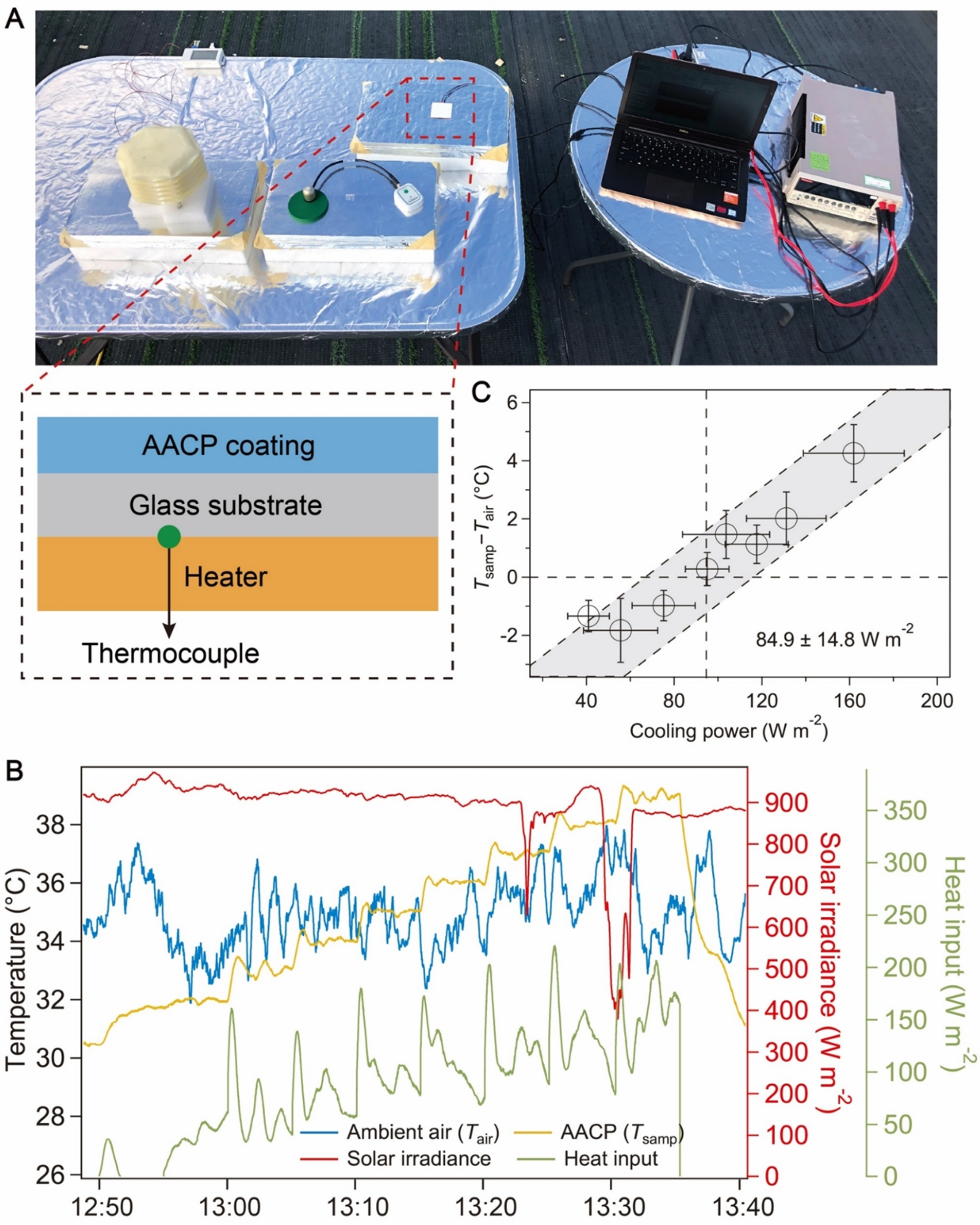

Supplementary Fig. 13. Net cooling power measurement — step-wise manner. (**A**) Photograph showing the setup for measuring the net cooling power. Insets showing the heater setup in side-view. (**B**) The obtained real-time temperature and heat input data at noon on a rooftop in Chengdu, China (September 29th, 2021). (**C**) Calculated temperature differences ($\Delta T = T_{samp} - T_{air}$) as a function of cooling power. When ΔT reaches zero, we obtain the cooling power as 84.9 ± 14.8 W m^{-2}.

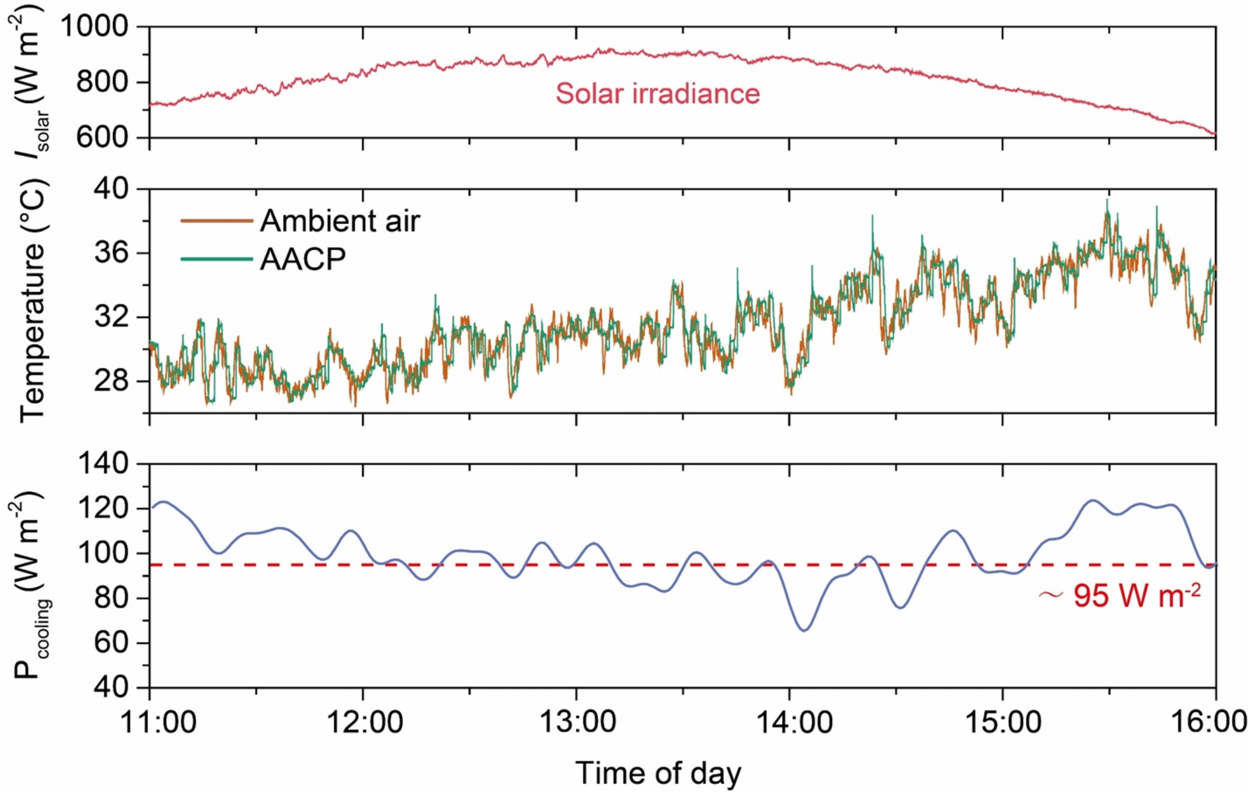

Supplementary Fig. 14. Net cooling power measurement — close-tracking manner. The obtained solar irradiance, real-time ambient temperature and AACP coating temperature heated by a PID controlled heater, from 11 AM to 4 PM, on a rooftop in Chengdu, China (April 20th, 2022). We obtained the cooling power as about 95 W m^{-2}.

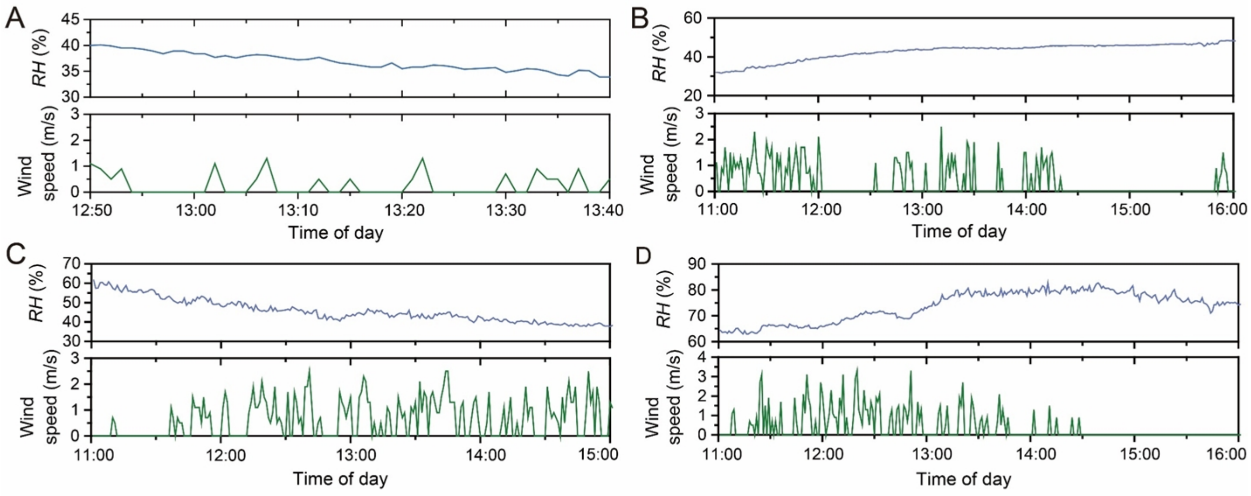

Supplementary Fig. 15. Humidity and wind speed data on the day of the field tests. (**A**) September 29th, 2021, Chengdu, corresponding to the Supplementary Fig. 13B. (**B**) April 20th, 2022, Chengdu, corresponding to Supplementary Fig. 14. (**C**) August 23rd, 2021, Chengdu, corresponding to the Fig. 2b in the main text, and Supplementary Fig. 17B. (**D**) May 25th, 2021, Chengdu, corresponding to the Fig. 4e in the main text.

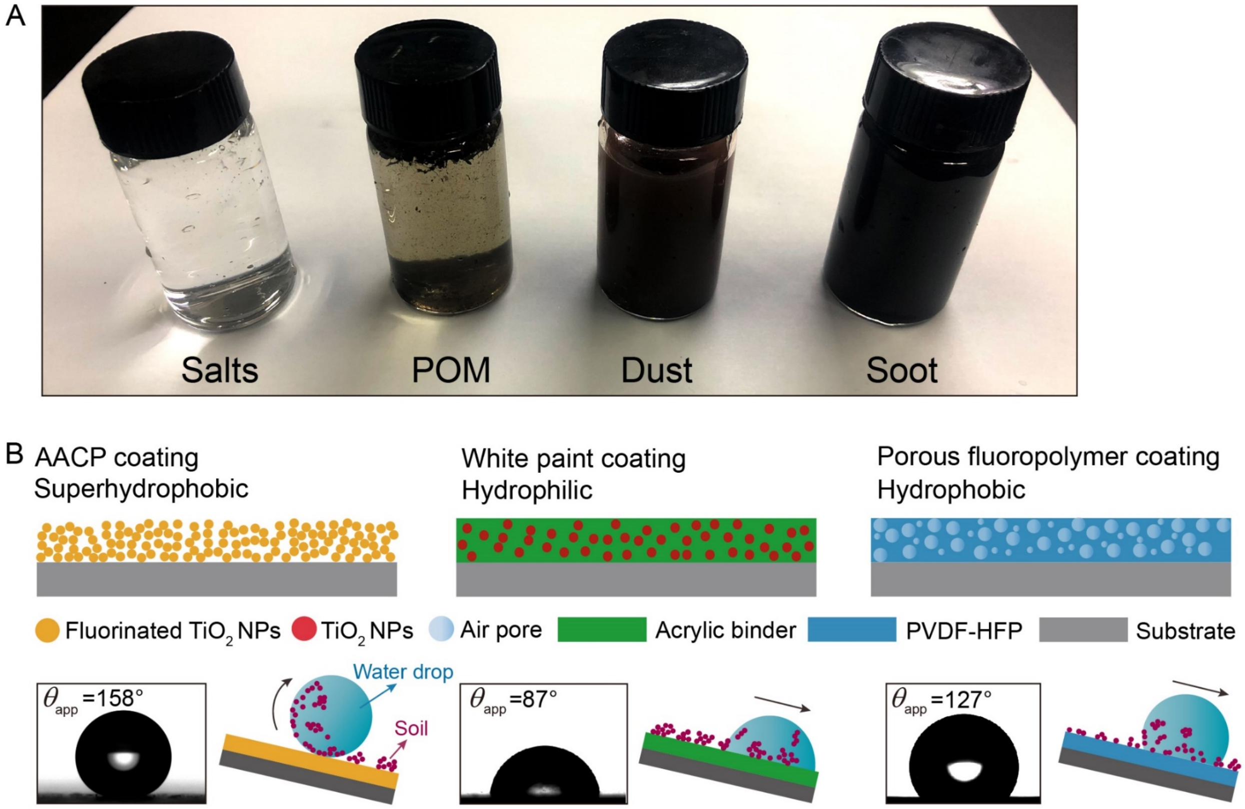

Supplementary Fig. 16. Soiling agents and soiling mechanisms on different coating surfaces. (**A**) Four soiling agents composing the simulated natural soiling agent. (**B**) Soiling mechanisms on different coating surfaces. Only superhydrophobicity can endow the surface self-cleaning ability when water drop rolls on the surface. The modest hydrophobic surface from porous PVDF-HFP cannot retain a clean surface, thus not ideal for durable SDRC against environmental aging.

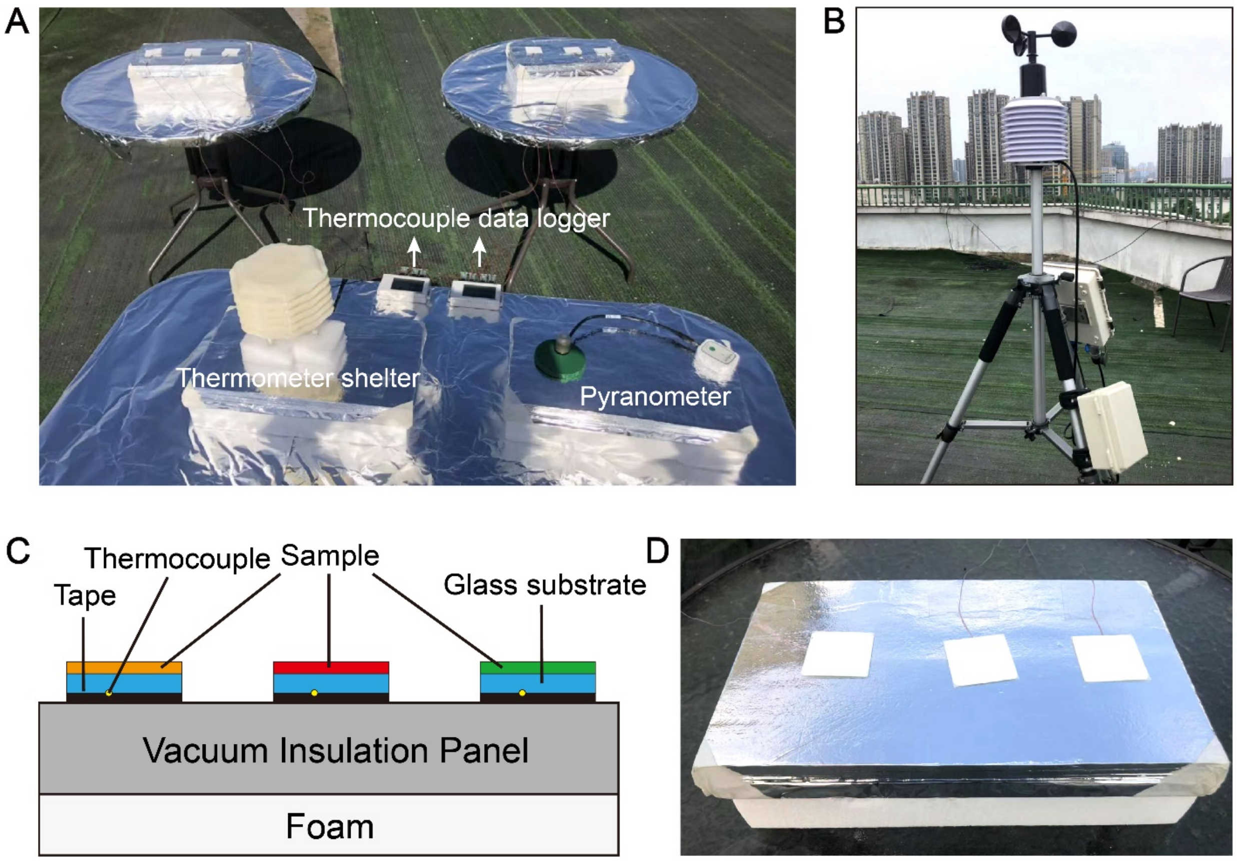

Supplementary Fig. 17. Field test setup for coating temperature measurements. (**A**) Photograph showing the experimental setup on a rooftop in Chengdu of China for monitoring the coating samples' temperatures under direct sunlight. (**B**) Photograph of a weather station. (**C**) Schematic showing the side-view of the experimental setup. (**D**) Top-view photograph showing the coatings tested without any convection shield.

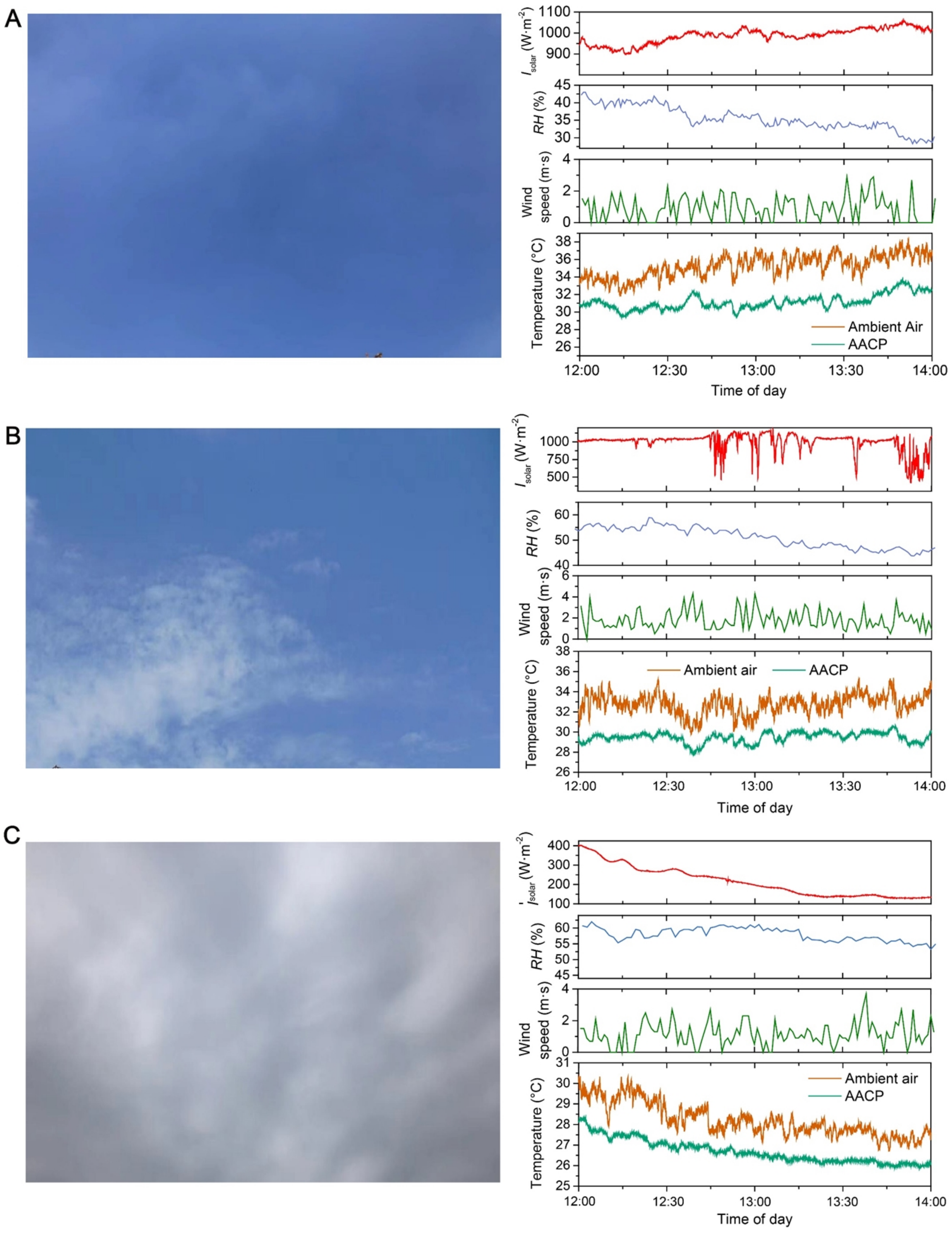

Supplementary Fig. 18. AACP coating cooling performances under different weather conditions. Photos of different cloud conditions in Chengdu and corresponding temperature measurements, relative humidity (*RH*) and solar irradiance (I_{solar}). (**A**) Clear sky (May 31st, 2021). (**B**) Partly cloudy (June 04th, 2021). (**C**) Overcast (June 03rd, 2021). ΔT ($\Delta T = T_{samp} - T_{air}$) was approximately −3.6, −3.2 and −1.5 °C, respectively.

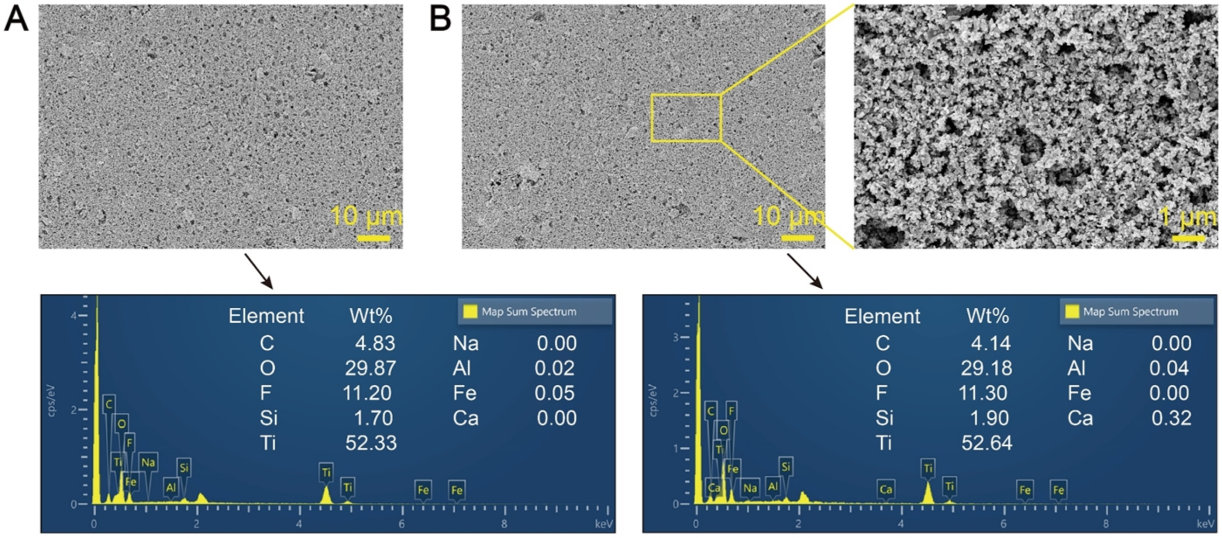

Supplementary Fig. 19. SEM images and corresponding EDS analysis of AACP coating surface. (**A**) and (**B**) are top view images of AACP coating before and after ASTM D7897-18 standard soiling test. Since the simulated soiling agents are composed of salts, POM, dust and soot, the elemental composition of Na, Al, Fe and Al could be used as indication of cleanness of the AACP coating surface, from which little change was observed showing the AACP's excellent anti-soiling function.

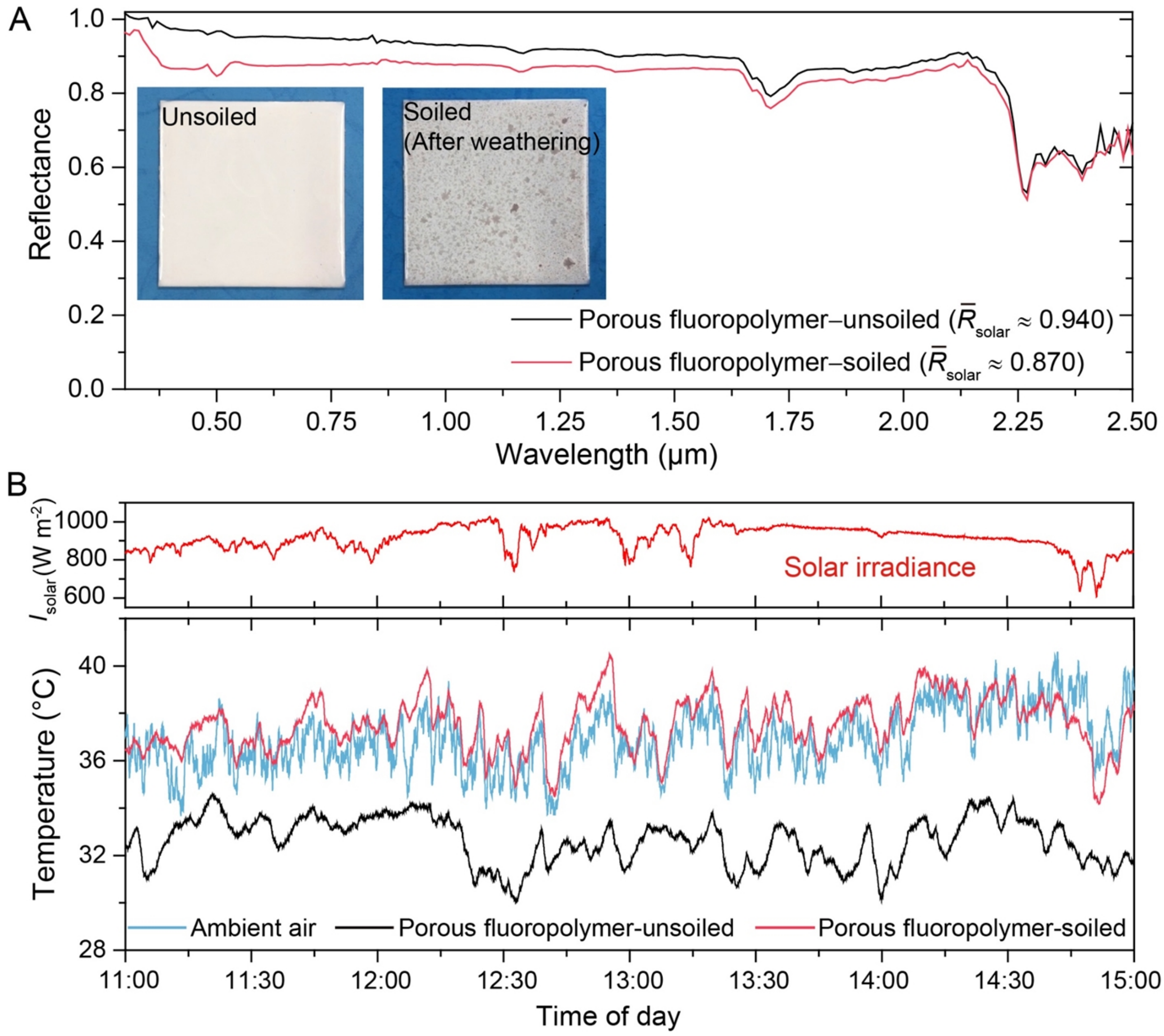

Supplementary Fig. 20. ASTM D7897-18 standard Soiling effect on porous PVDF-HFP coating. (**A**) Reflectance curves of porous PVDF-HFP coating before and after simulated 3 years of standard soiling test, showing a decline of 7.45% of the original $\bar{R}_{solar}$ (from 0.940 to 0.870). Visual appearances showing the soiling effect on the porous PVDF-HFP coating. (**B**) The ΔT ($\Delta T = T_{samp} - T_{air}$) data for porous PVDF-HFP coating before/after soiling were approximately −4.2/0.7 °C. The temperature measurements were taken at the same time with all the other samples shown in the main text, Fig. 3B (Chengdu, August 23rd, 2021).

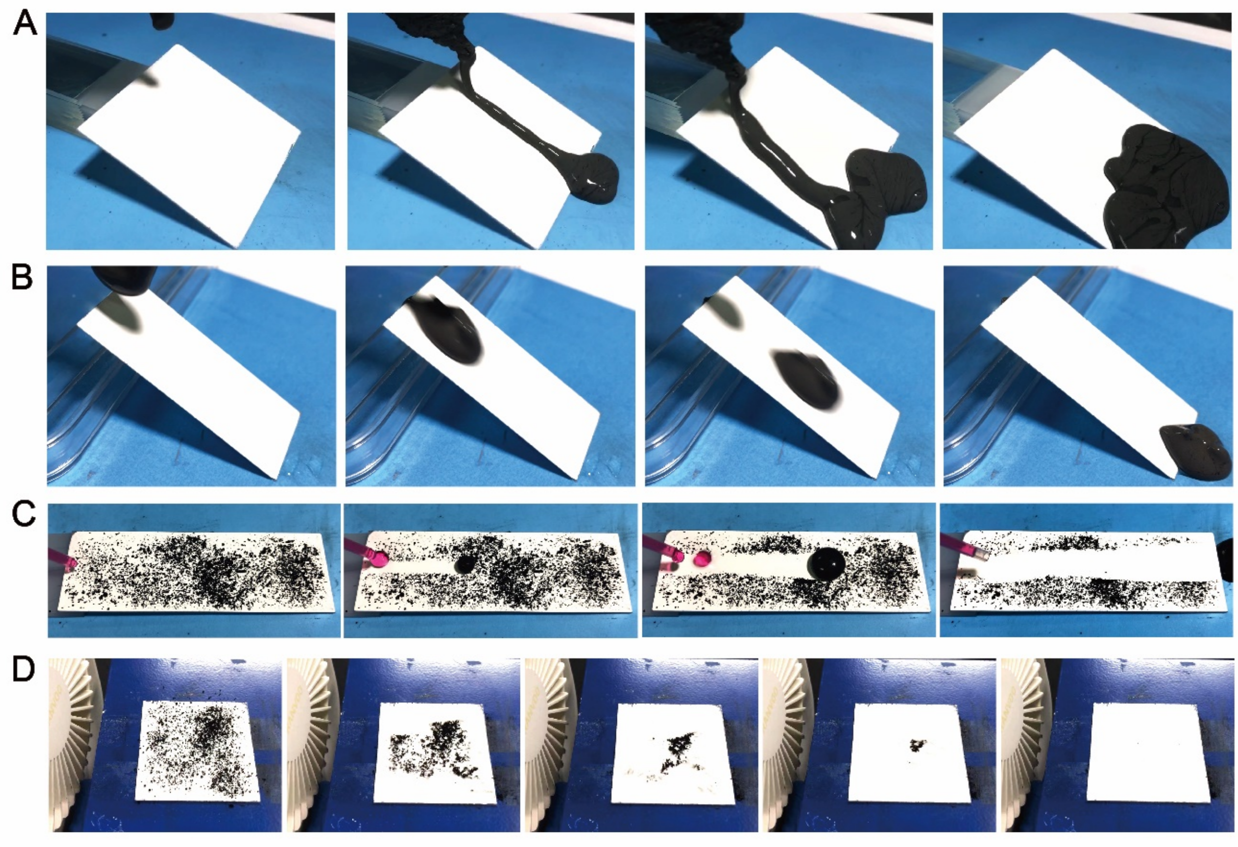

Supplementary Fig. 21. Soiling effects of mud and MnO_2 particles on AACP coating. Photographs showing (A) low viscous and (B) high viscous mud dripped and flowed off the AACP coating. (C) Water droplet driven cleaning effect and (D) wind blow driven cleaning effect against the soiling of MnO_2 particles.

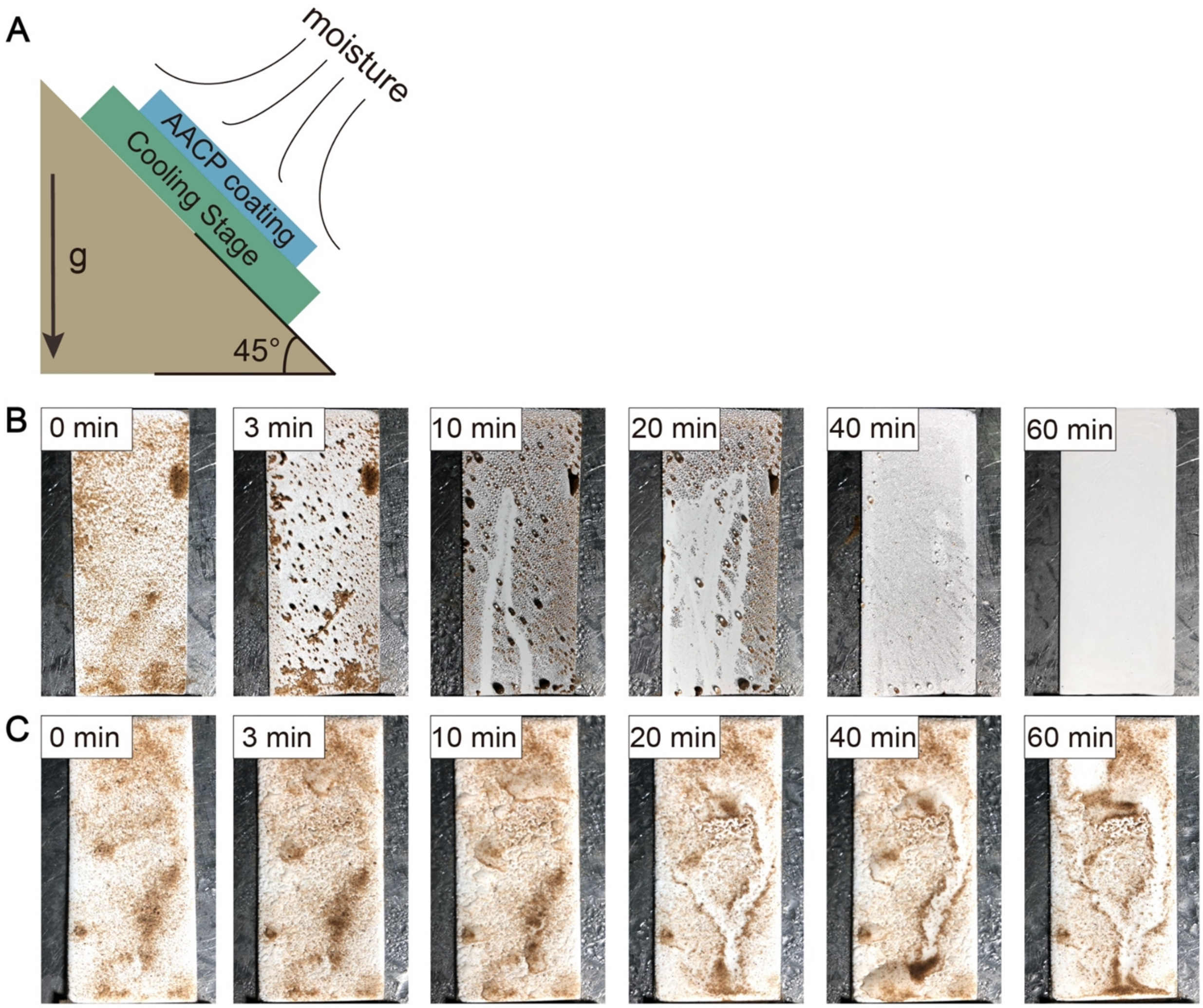

Supplementary Fig. 22. Soiling of sand and moisture driven cleaning effect. (**A**) Schematic showing the moisture driven cleaning apparatus. A tilted cooling stage was used to induce water condensation. Photographs presenting series of time evolution showing the sand soiling and water condensation driven cleaning effect for (**B**) AACP and (**C**) white paint coating. The moisture stopped at 40 min. After 20 minutes (60 min marked photos), our AACP coating showed completely dry surface with no visible sand left on it.

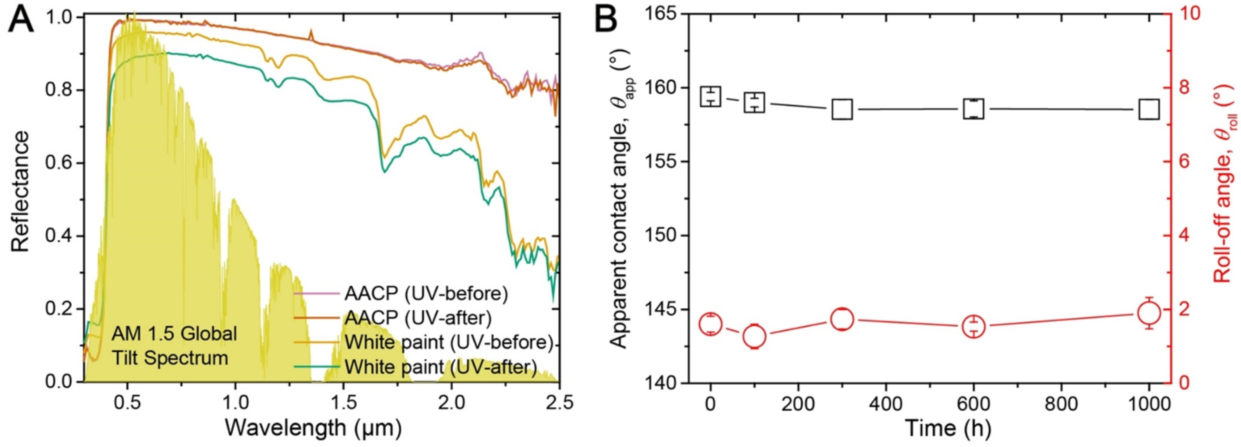

Supplementary Fig. 23. UV weathering effects on AACP and white paint coatings. 1,000 h UV with 0.89 W m^{-2} at 340 nm and 60 °C were implemented on AACP and white paint coatings. (**A**) Effect on the solar reflectance of the coatings. The calculated $\bar{R}_{solar}$ data were shown in the main text, Fig. 3D (**B**) Effect on the wetting property of AACP coating. Apparent contact angle (θ_{app}) stayed above 155°. Roll-off angle (θ_{roll}) stayed below 2°.

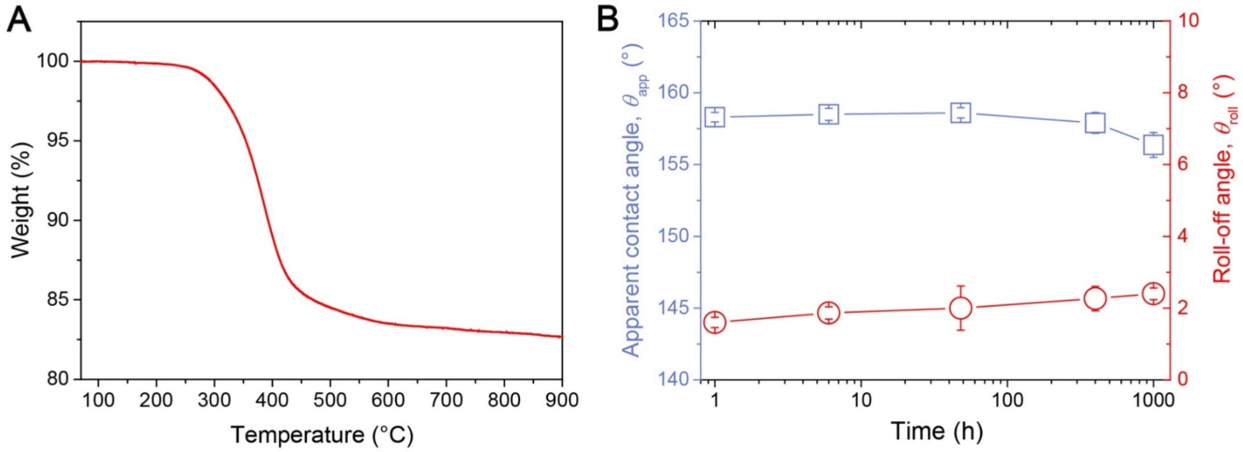

Supplementary Fig. 24. Thermal stability of AACP. (**A**) The TGA curve of the fluorinated TiO_2 nanoparticles showing they are highly stable at about 200 °C. (**B**) The effect of 1,000 h 100 °C on the AACP coating with adhesives. θ_{app} stayed above 155° and θ_{roll} stayed below 3°.

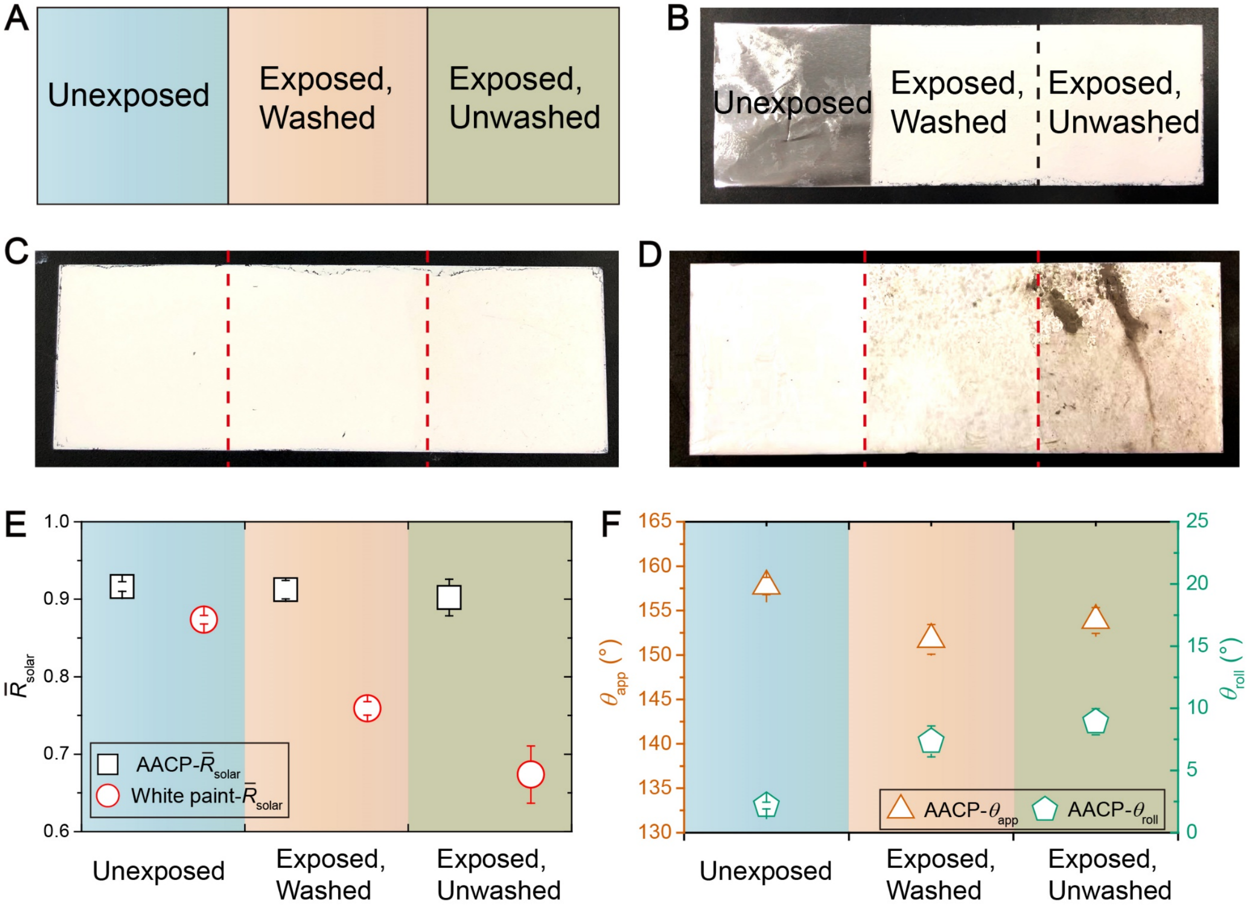

Supplementary Fig. 25. Real-world aging (exposure in Xi'an, dry and hot climate, from May 1st to November 30th, 2021). (A) Schematic showing the three regions corresponding to different exposure ways as: unexposed/(exposed, washed)/(exposed, unwashed). (B) Photograph showing the AACP coating wrapped in aluminum foil. (C) Retrieved AACP coatings after aging showing exceptional cleanness. (D) Retrieved white paint coatings after aging showing darkened surface even after wash. (E) $\bar{R}_{solar}$ of AACP coatings for the three regions are 0.916/0.912/0.903, showing a decrease of 0.4% and 1.4%. $\bar{R}_{solar}$ of white paint coatings are 0.874/0.759/0.674, showing a decrease of 13.2% and 22.9%. (F) After aging, for AACP coatings, the θ_{app} stayed above 150° and θ_{roll} stayed below 10°.

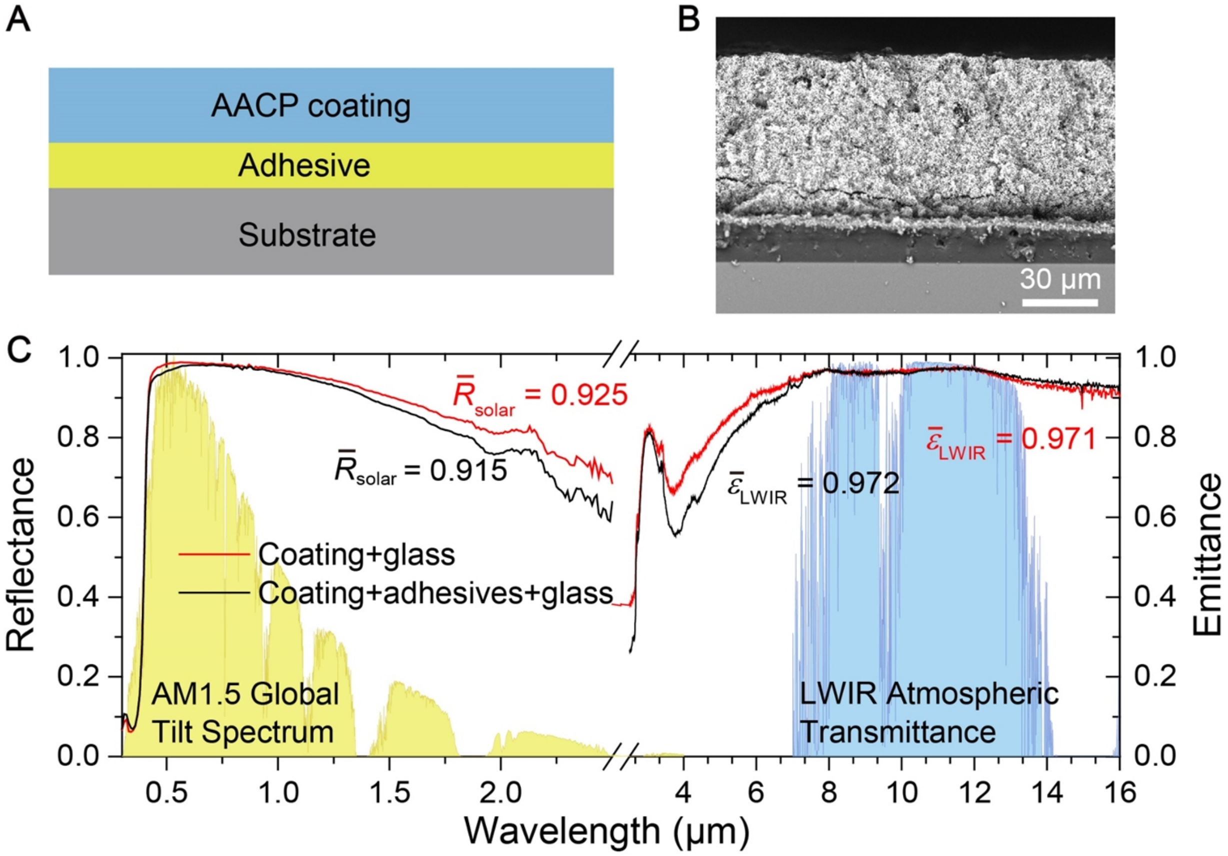

Supplementary Fig. 26. "Paint + adhesive" reinforced AACP coating. (**A**) Schematic showing the structure of "AACP + adhesive". (**B**) SEM image showing AACP coating on a layer of adhesive. (**C**) The reflectance and emittance curves showing the effect of adhesive on AACP coating's optical properties is negligible.

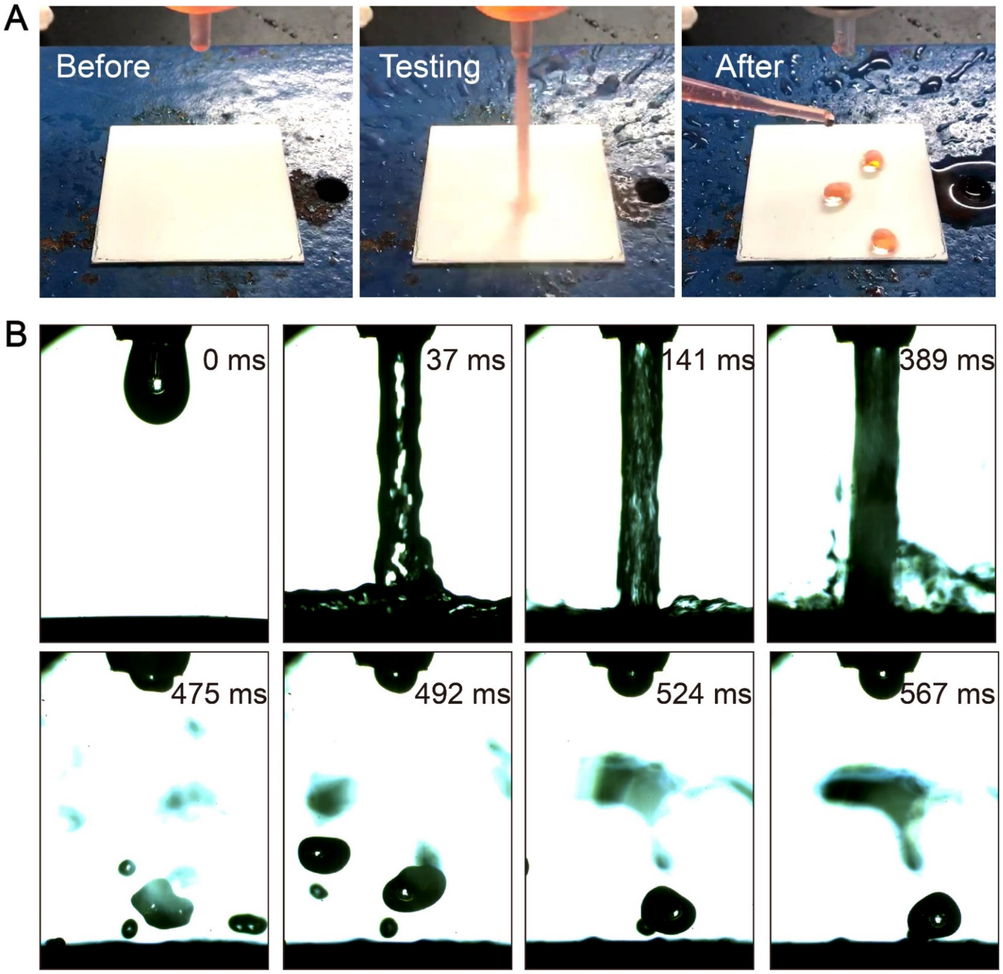

Supplementary Fig. 27. High-speed water jet test. Photographs (**A**) and high-speed camera images (**B**) of the high-speed water jet impact on AACP. 10 mL of water was jetted from a 2 mm diameter needle within 400 ms as one test cycle, with an average speed of 8 m s^{-1}. This speed is similar to the speed of raindrops in a rainstorm (≈ 9 m s^{-1}). Sliding and rebounding of droplets after jetting demonstrate exceptional water repellency of AACP coating.

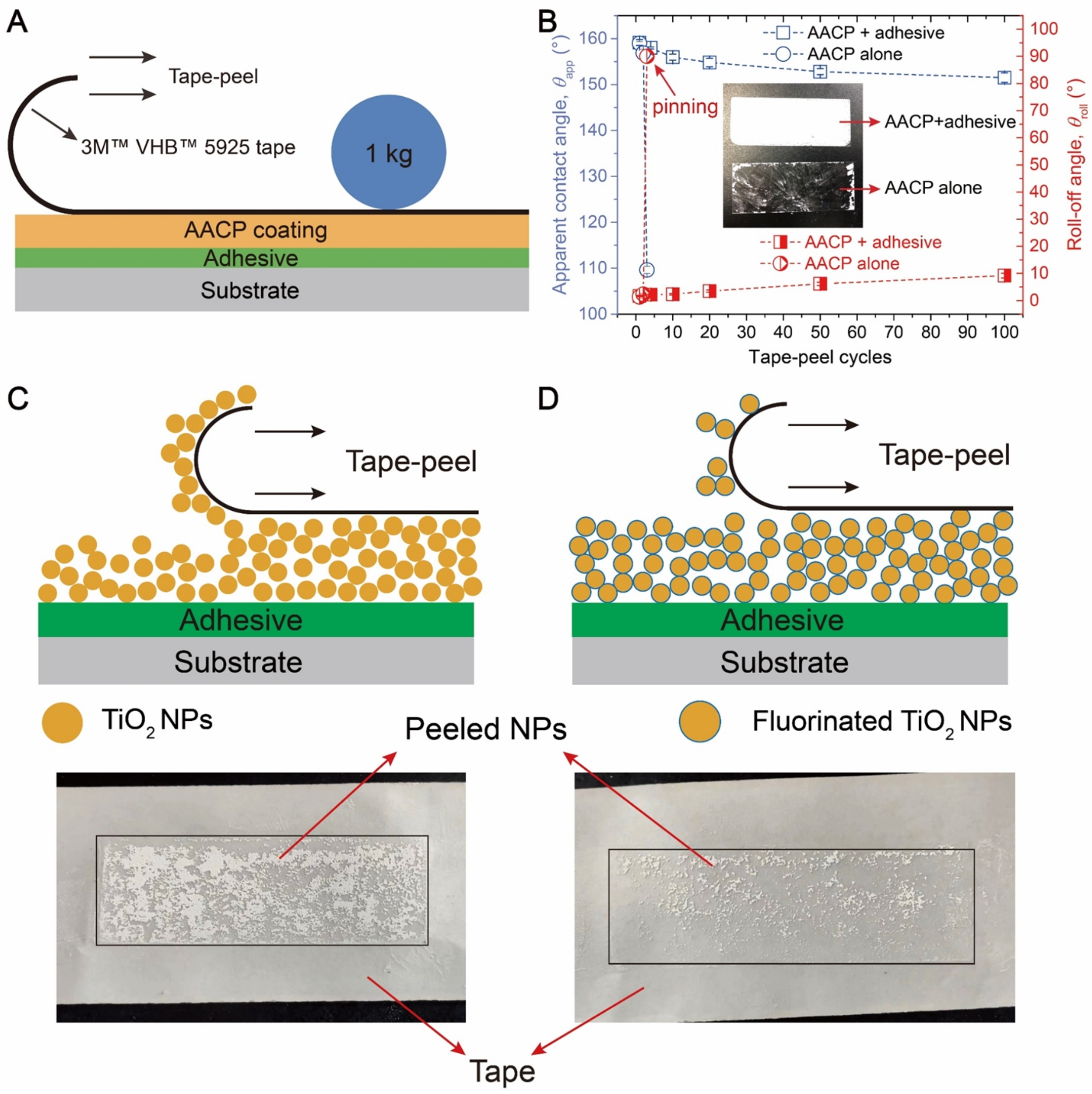

Supplementary Fig. 28. Tape-peel test. (**A**) Schematic illustrating tape-peel test. (**B**) After 100 cycles of tape-peel, θ_{app} stayed abovge 150° and θ_{roll} stayed below 10°. The inset gives the visual appearance of the "AACP + adhesive" after 100 cycles of tape-peel and "AACP alone" after 3 cycles of tape-peel. Without adhesive, the coating layer was completely peeled off from the substrate after just 3 cycles of tape-peel. While with adhesive, the AACP coating's wetting properties were almost intact thanks to the self-similar morphology of random packed TiO_2 NPs. Besides adhesive, the fluorination also plays an important role of improving the overall coating robustness. After one cycle of tape-peel, compared with (**C**) the non-fluorinated TiO_2 NPs, the amount of peeled NPs (**D**) by the tape is much less thanks to the bonding effect from the fluorination layer of TiO_2 NPs.

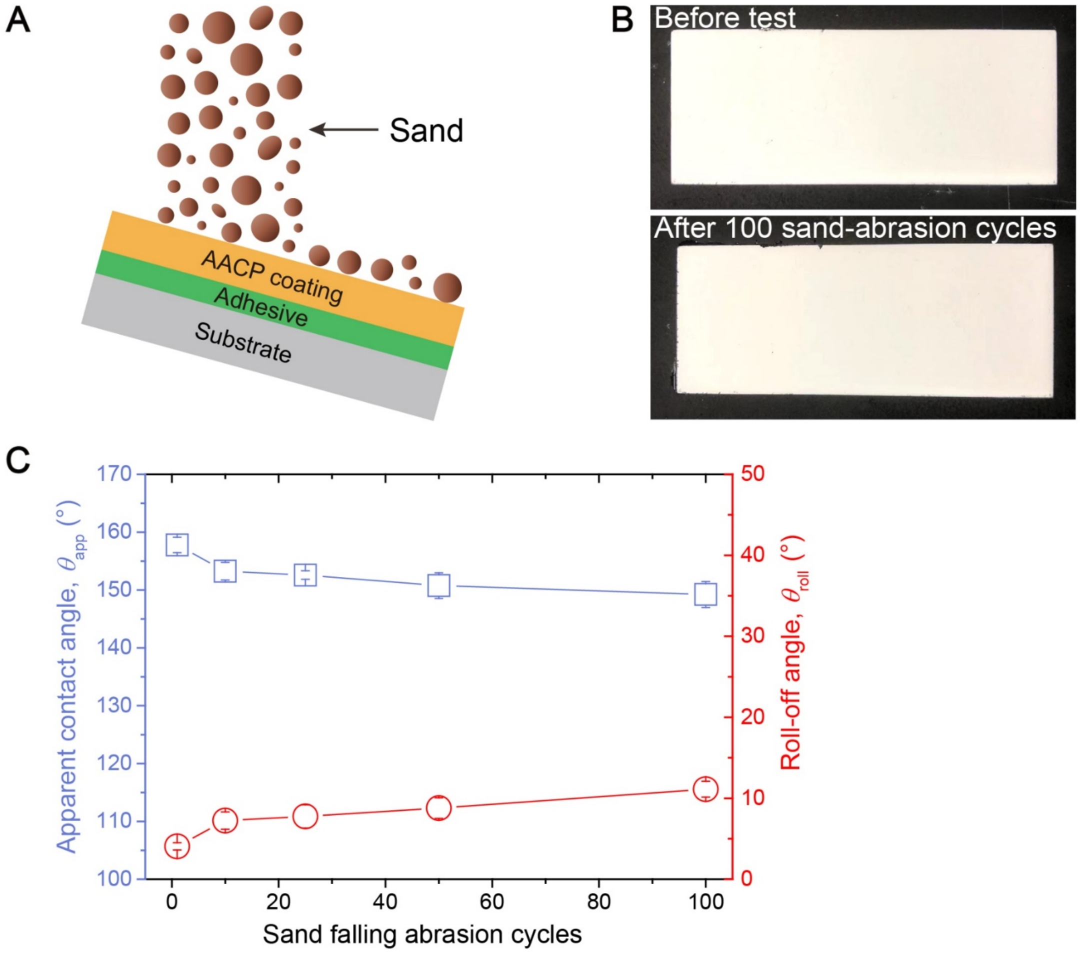

Supplementary Fig. 29. Sand falling abrasion test. (**A**) Schematic showing the sand falling abrasion test setup. (**B**) Visual appearance showing the almost intact surface (24 × 60 mm) after 100 cycles of sand falling. (**C**) Our AACP coating remained to be superhydrophobic even after 100 cycles of sand falling abrasion test.

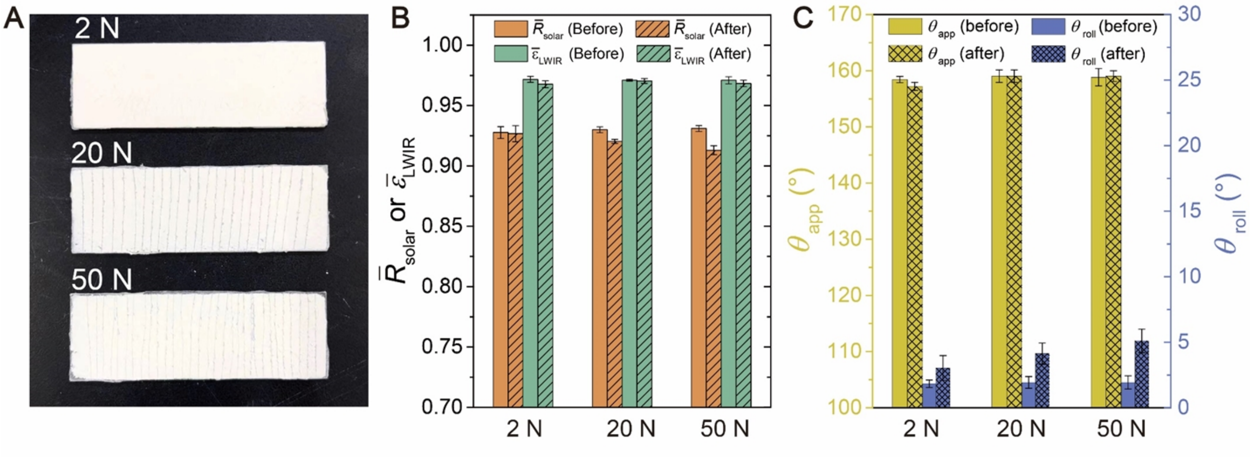

Supplementary Fig. 30. Scratch test. (**A**) Visual appearance of AACP coating against scratches under various load of forces. (**B**) and (**C**) demonstrate optical and wetting properties before and after scratches, showing great resistance of AACP coating against scratches. We found that the $\bar{R}_{solar}$ of AACP coating of scratch load of 2 N, 20 N and 50 N declined only by 0.1%, 1.0% and 1.9% respectively compared with the untested ones (Supplementary Table 2). The θ_{app} all stayed above 150° and θ_{roll} stayed below 5°.

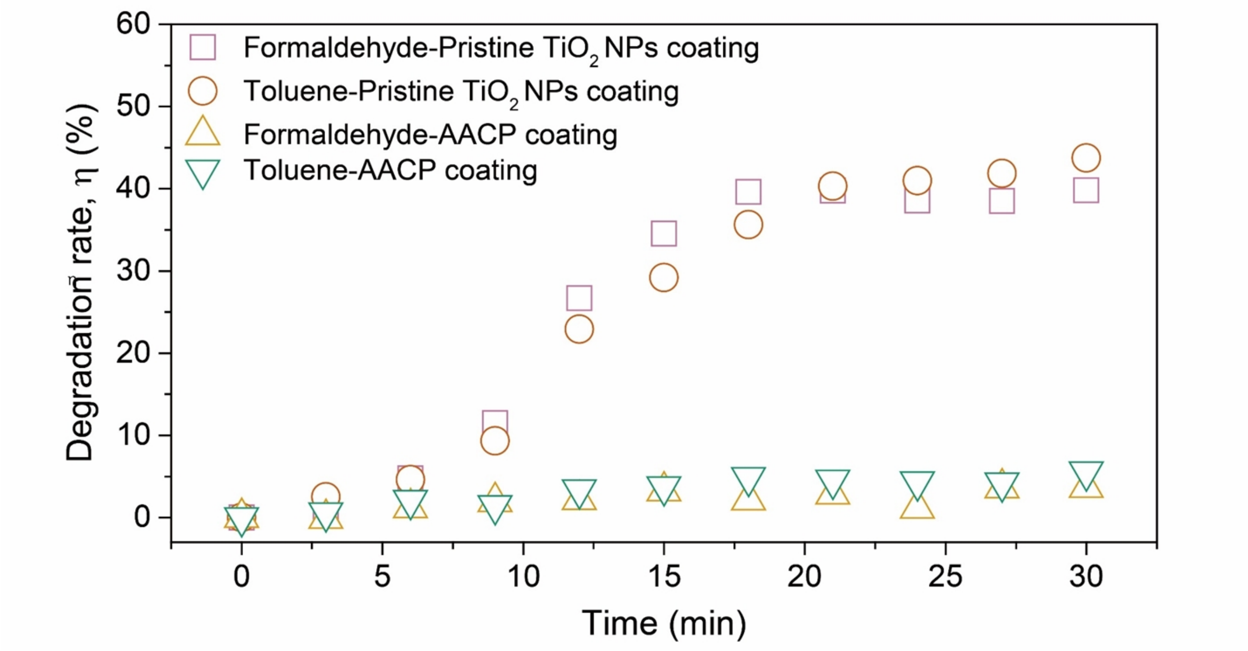

Supplementary Fig. 31. Photocatalysis effect of AACP coating. The result show that less than 5% of formaldehyde and toluene was removed by AACP coating during 30 min of UV illumination period. For pristine TiO_2 nanoparticles, the degradation rate is generally more than 40%. This result indicates that the AACP coating has little photocatalytic activity. We attribute that the non-photocatalytic activity of AACP to the hydrophobic PFOTS layer on the surface of TiO_2, which prevents water and oxidant directly absorbed on the surface of TiO2 to participate in a photocatalytic oxidation reaction.

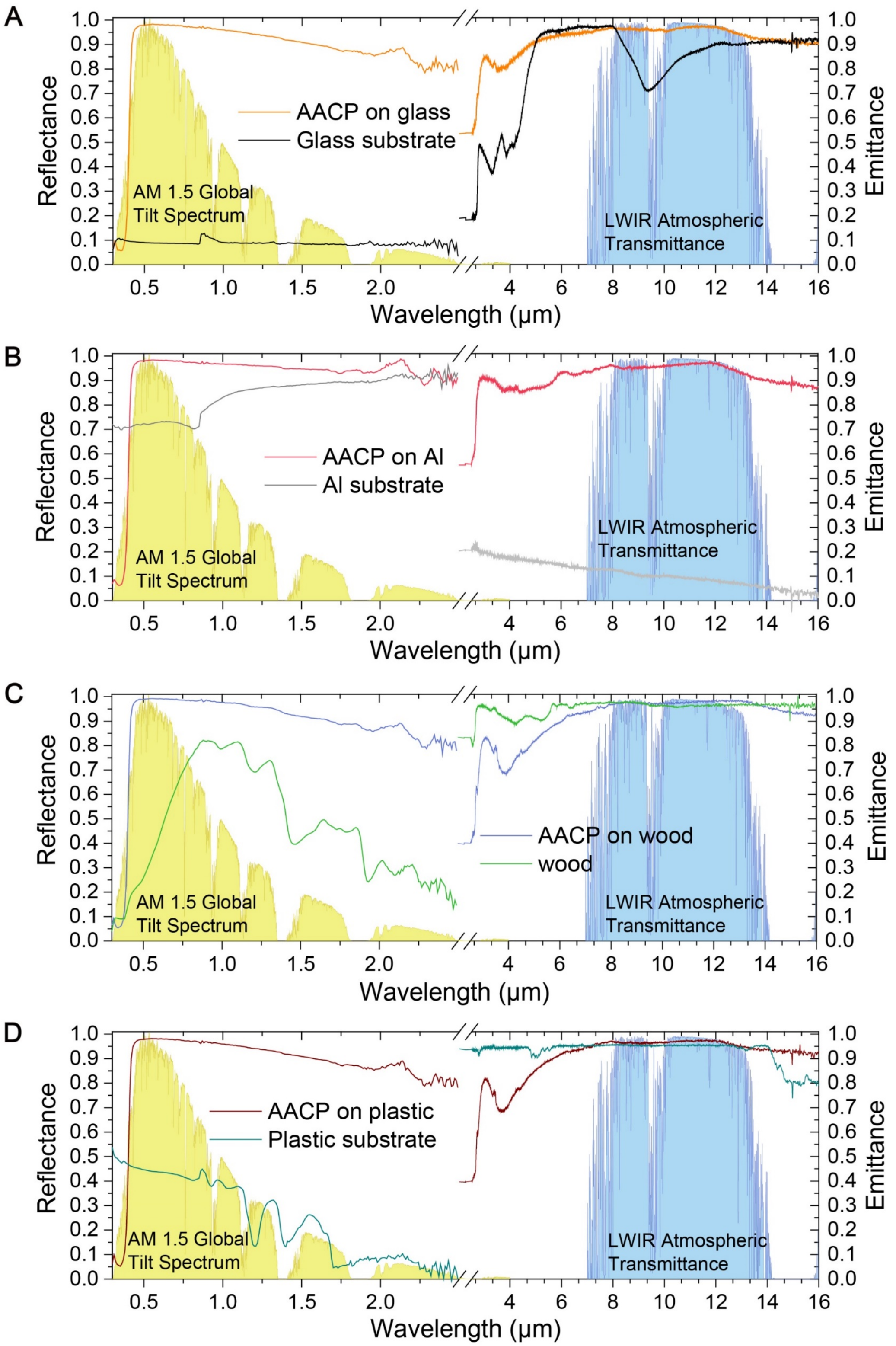

Supplementary Fig. 32. AACP on diverse substrates. The reflectance and emittance curves for diverse substrates and AACP coated ones: (**A**) glass, (**B**) metal (aluminum, Al), (**C**) wood (basswood), (**D**) plastic (PMMA). The calculated $\overline{R_{\text{solar}}}$ and $\bar{\varepsilon}_{\text{LWIR}}$ are displayed in Table 3.

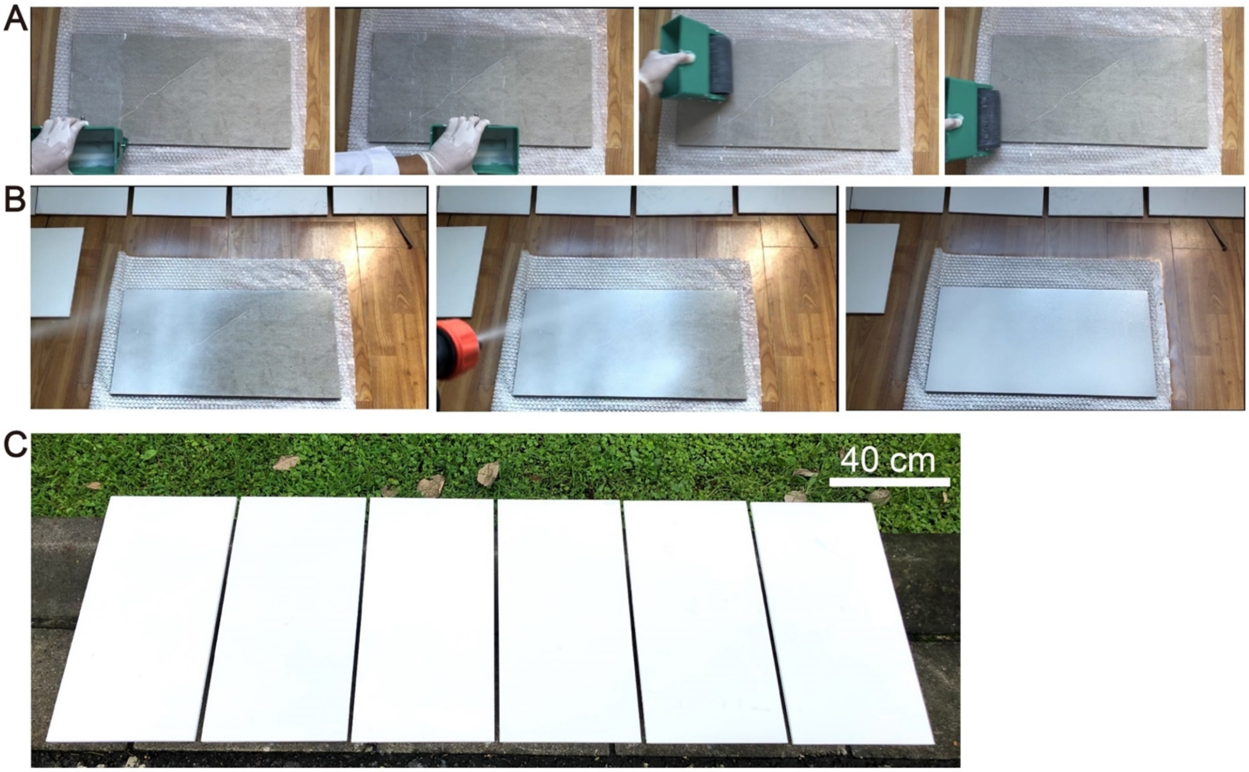

Supplementary Fig. 33. Fabrication of AACP coated outdoor wall tiles. (**A**) The process of implementing adhesive. (**B**) Spray coating of AACP on the tiles. (**C**) Half a dozen of AACP coated wall tiles (40 × 80 cm).

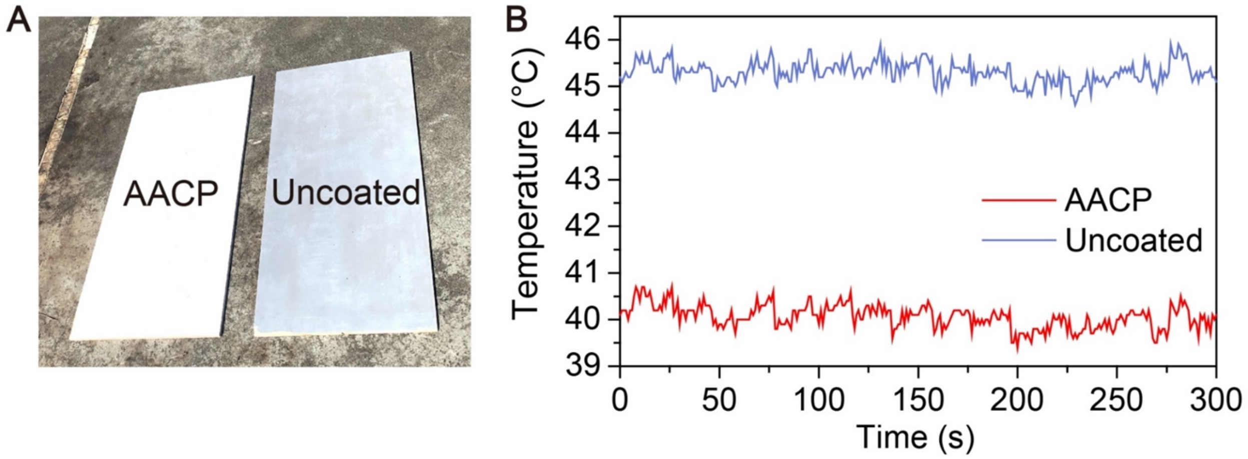

Supplementary Fig. 34. Demonstration of the AACP coated wall tile's cooling ability. (**A**) Photograph showing the visual appearance difference between AACP coated and uncoated wall tiles. (**B**) Temperature data (corresponding to the infrared image shown in the main text, Fig. 4D) showing excellent cooling ability of our AACP coating.

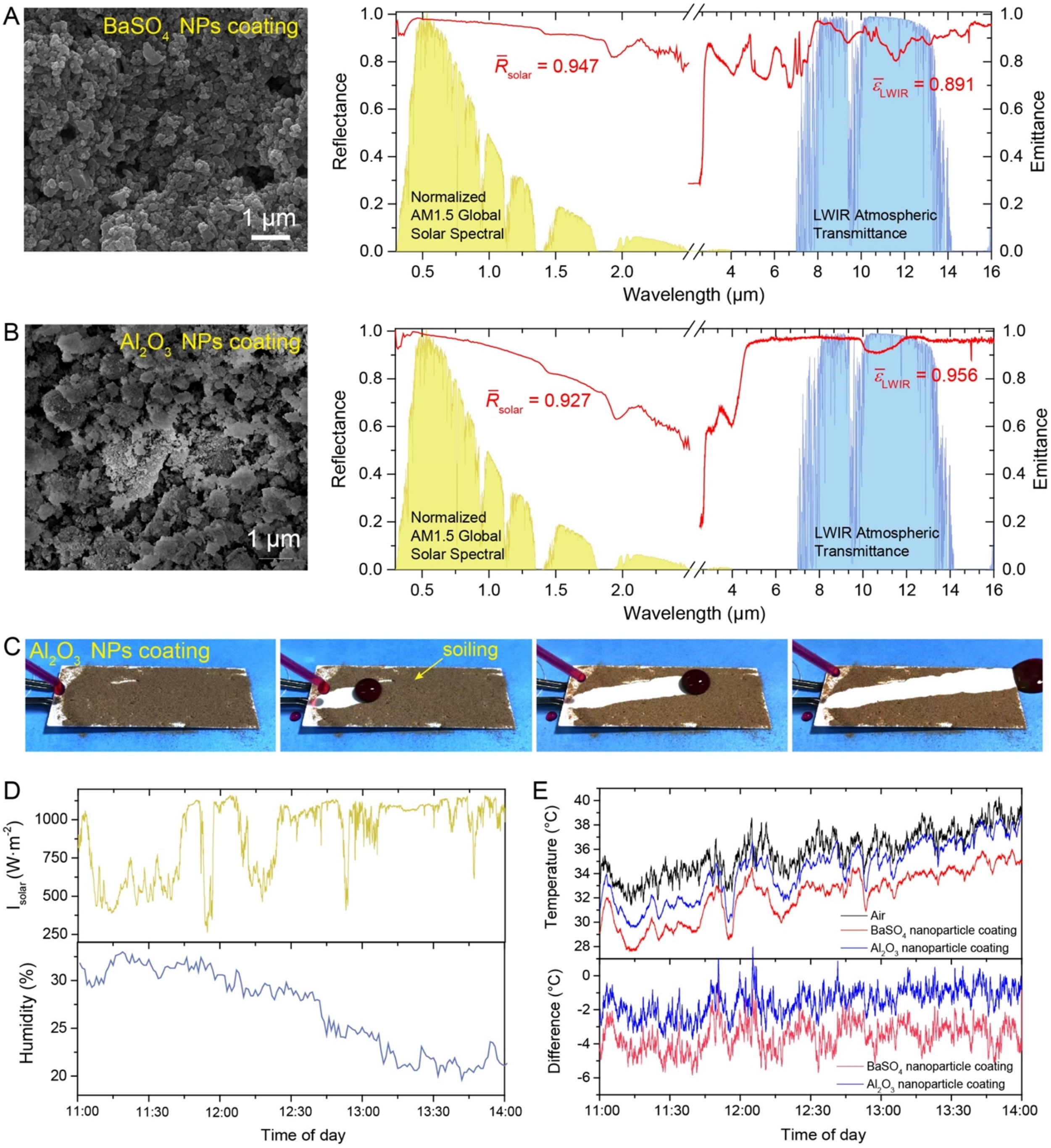

Supplementary Fig. 35. $BaSO_4$ and Al_2O_3 nanoparticles for SDRC. (**A**) SEM image of $BaSO_4$ coating surface and optical property. (**B**) SEM image of Al_2O_3 and corresponding optical property. (**C**) Demonstration of self-cleaning on Al_2O_3 nanoparticles coating. (**D**) The weather condition of the field test (June 5th, 2021, Chengdu). (**E**) Temperature monitoring of ambient air and coatings. ΔT ($\Delta T = T_{samp} - T_{air}$) was approximately −3.7 and −1.8 °C for $BaSO_4$ and Al_2O_3 nanoparticle coatings respectively.

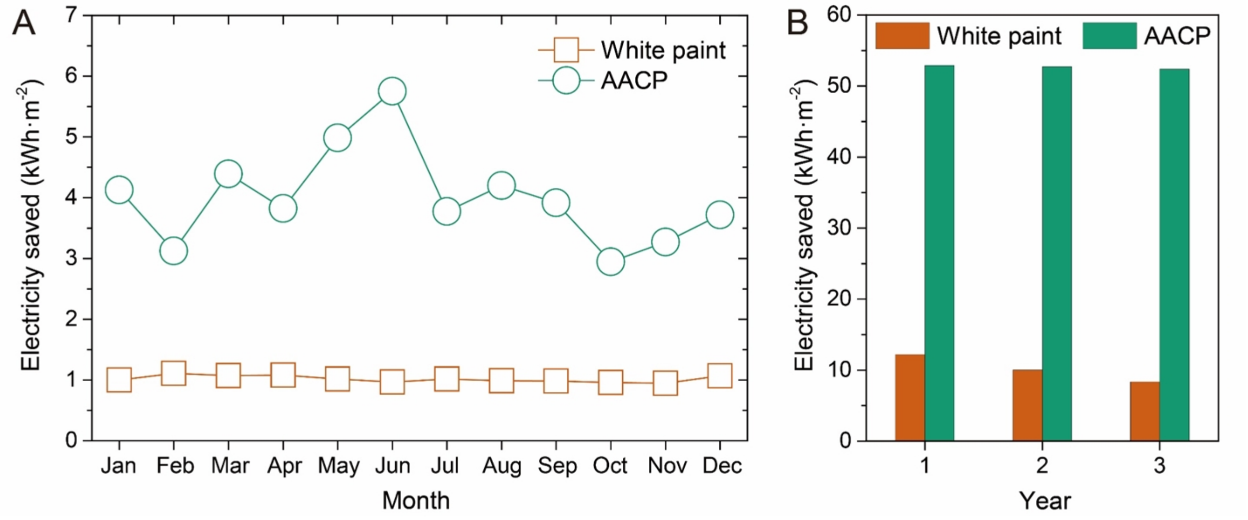

Supplementary Fig. 36. Comparison of electricity saved by implementing AACP and white paint on the roof of medium commercial reference building based on EnergyPlus simulation. (**A**) At the first year, electricity savings for each month. (**B**) Comparison of electricity saving for a year in a period of three years. Due to environmental aging, the electricity saving for white paint decreases each year from 12.2 to 8.4 kWh m^{-2}, while AACP renders robust energy saving performance above 50 kWh m^{-2}.

Supplementary Table 1. Summary of additional soiling tests.

<table>
<tr><th rowspan="2" colspan="3">Additional soiling tests</th><th colspan="2">$\bar{R}_{solar}$</th></tr>
<tr><th>Before</th><th>After</th></tr>
<tr><td rowspan="3">Mud soiling</td><td colspan="2">Coal ash weight ratio (%)</td><td>/</td><td>/</td></tr>
<tr><td colspan="2">50</td><td>0.922</td><td>0.914</td></tr>
<tr><td colspan="2">80</td><td>0.928</td><td>0.923</td></tr>
<tr><td rowspan="7">MnO_2 particles soiling</td><td>Diameter of MnO_2 particles (D)</td><td>Cleaning method</td><td>/</td><td>/</td></tr>
<tr><td rowspan="2">D < 1 μm</td><td>Wash</td><td>0.933</td><td>0.919</td></tr>
<tr><td>Wind blow</td><td>0.929</td><td>0.901</td></tr>
<tr><td rowspan="2">1 < D < 10 μm</td><td>Wash</td><td>0.934</td><td>0.923</td></tr>
<tr><td>Wind blow</td><td>0.929</td><td>0.902</td></tr>
<tr><td rowspan="2">D > 10 μm</td><td>Wash</td><td>0.931</td><td>0.927</td></tr>
<tr><td>Wind blow</td><td>0.925</td><td>0.921</td></tr>
</table>

Supplementary Table 2. Summary of various tests showing excellent mechanical stability of AACP coatings.

Test	Description	$\bar{R}_{\mathrm{solar}}/\bar{\varepsilon}_{\mathrm{LWIR}}$	
		**Before**	**After**
Accelerated thermal stability test	100 °C, 1,000 h.	0.926/0.975	0.925/0.971
High-speed water jet impact test	100 times of high-speed water jet impact (2 mm jet at 8 m s^{-1}, 10 mL of water each time).	0.918/0.969	0.914/0.969
Tape-peel test	100 times of tape-peel test (the 3M VHB 5925 tape was applied under uniform pressure by rolling a 1 kg roller once each time).	0.913/0.975	0.876/0.974
Sand falling abrasion test	100 times of sand falling at a height of 30 cm, 20 g of sand mass was used each time.	0.925/0.971	0.906/0.965
Scratch test with load of 2 N	A scratch was applied onto the AACP coating surface under a normal load of 2 N over a distance of 0.1 m at a constant scratch rate of 0.1 m s^{-1}	0.928/0.972	0.926/0.967
Scratch test with load of 20 N	A scratch was applied onto the AACP coating surface under a normal load of 20 N over a distance of 0.1 m at a constant scratch rate of 0.1 m s^{-1}	0.93/0.971	0.92/0.97
Scratch test with load of 50 N	A scratch was applied onto the AACP coating surface under a normal load of 50 N over a distance of 0.1 m at a constant scratch rate of 0.1 m s^{-1}	0.931/0.971	0.913/0.968

Supplementary Table 3. Summary of optical properties of AACP on diverse substrates.

**Materials**	$\bar{R}_{\text{solar}}/\bar{\varepsilon}_{\text{LWIR}}$
Glass	0.092/0.863
Metal (Al)	0.775/0.096
Wood	0.513/0.967
Plastic (PMMA)	0.389/0.962
Glass + AACP	0.922/0.972
Metal + AACP	0.931/0.969
Wood + AACP	0.923/0.973
Plastic + AACP	0.919/0.973

Supplementary Table 4. Recent works demonstrating radiative cooling with hydrophobicity.

Materials	$\bar{R}_{solar}$/ $\bar{\varepsilon}_{LWIR}$	θ_{app}/ θ_{roll}	Anti-soiling test	UV test	Real-world test	Reference
AACP	0.93/0.97	158°/2°	ASTM D7897-18	1,000 h	Over 6 months	Our work
Porous PVDF	0.96/0.97	110°/NA	Dye liquid	NA	One month	Science (*9*)
Porous PVDF and PVA	0.94/0.94	About 140°/NA	NA	NA	NA	ACS Nano (*10*)
Porous PMMA	0.95/0.98	156°/NA	NA	480 h	40 days	Nat. Commun. (*11*)
Delignified wood	0.96/0.9	150°/NA	NA	NA	NA	Science (*12*)
Nanoporous PE fibres	0.9/0.9	Hydrophobic/NA	NA	NA	NA	Nat. Sustain. (*13*)
Multilayer of SiO_2 spheres, SiO_2 film, SiO_xN_y backed by silver film	0.96/0.95	129°/NA	Dust and moisture	720 h	NA	Adv. Mater. (*14*)
TiO_2, polymer binder, silica microspheres and fluorescent pigment	0.93/0.96	113°/NA	Ash	960 h	NA	Adv. Mater. (*15*)
Cellulose and PTFE coating	0.93/0.9	165°/6°	Dye and oil	30 days	4 weeks	ACS Appl. Mater. Interfaces (*16*)
Porous ceramic and PTFE	0.88/0.92	160°/6.8°	Dye liquid	NA	100 days (no optical data reported)	ACS Appl. Mater. Interfaces (*17*)

Supplementary Table 5. Comparison of AACP and two other white paint formulations cost.

Product	Market Price	Recommended Painting area	Price per square meter	Brand or website
Our work: AACP	$17-$19.6/Liter (Ethanol: $2-$2.5/Liter; TiO_2: $50-$56/kg; Trichloro(1H,1H,2H,2H-tridecafluoro-n-octyl)silane: $2-$2.3/g)	3-4 m^2/Liter	$4.25-$6.53	Aladdin Inc. for TiO_2 and ethanol, HWRK Chem Inc. for fluorosilane
Sherwin-williams: Solo Interior/Exterior Acrylic Paint (extra white, product number A74W00051)	$19.31-$20.95/Liter	4-6 m^2/Liter	$3.22-$5.24	Sherwin-williams Co.
Majic PAINTS Interior/Exterior Satin Paint, White.	$29.33/Liter	5-10 m^2/Liter	$2.93-$5.87	Amazon.com

Supplementary References

1. M. Kerker, *The scattering of light and other electromagnetic radiation*, (Academic Press, New York, 1969)
2. P. Laven, Mieplot. http://www.philiplaven.com/mieplot.htm
3. J. R. DeVore, Refractive indices of rutile and sphalerite. *J. Opt. Soc. Am.* **41**, 416-419 (1951). doi:10.1364/josa.41.000416
4. J. Kischkat, S. Peters, B. Gruska, M. Semtsiv, M. Chashnikova, M. Klinkmüller, O. Fedosenko, S. Machulik, A. Aleksandrova, G. Monastyrskyi, Y. Flores, W. Ted Masselink, Mid-infrared optical properties of thin films of aluminum oxide, titanium dioxide, silicon dioxide, aluminum nitride, and silicon nitride. *Appl. Opt.* **51**, 6789-6798 (2012). doi:10.1364/ao.51.006789
5. K. L. Johnson, K. Kendall, A. D. Roberts, D. Tabor, Surface energy and the contact of elastic solids. *Proc. R. Soc. Lond. A* **324**, 301-313 (1971). doi:10.1098/rspa.1971.0141
6. F. Geyer, M. D'Acunzi, A. Sharifi-Aghili, A. Saal, N. Gao, A. Kaltbeitzel, T. F. Sloot, R. Berger, H. J. Butt, D. Vollmer, When and how self-cleaning of superhydrophobic surfaces works. *Sci. Adv.* **6**, eaaw9727 (2020). doi:10.1126/sciadv.aaw9727
7. D. Wang, Q. Sun, M. J. Hokkanen, C. Zhang, F.-Y. Lin, Q. Liu, S.-P. Zhu, T. Zhou, Q. Chang, B. He, Q. Zhou, L. Chen, Z. Wang, R. H. A. Ras, X. Deng, Design of robust superhydrophobic surfaces. *Nature* **582**, 55-59 (2020). doi:10.1038/s41586-020-2331-8
8. J. S. Higgins, H. C. Benoit, *Polymers and neutron scattering*, (Oxford University Press, New York, 1994)
9. J. Mandal, Y. Fu, A. C. Overvig, M. Jia, K. Sun, N. N. Shi, H. Zhou, X. Xiao, N. Yu, Y. Yang, Hierarchically porous polymer coatings for highly efficient passive daytime radiative cooling. *Science* **362**, 315-319 (2018). doi:10.1126/science.aat9513
10. H. Zhong, Y. Li, P. Zhang, S. Gao, B. Liu, Y. Wang, T. Meng, Y. Zhou, H. Hou, C. Xue, Y. Zhao, Z. Wang, Hierarchically hollow microfibers as a scalable and effective thermal insulating cooler for buildings. *ACS Nano* **15**, 10076-10083 (2021). doi:10.1021/acsnano.1c01814
11. T. Wang, Y. Wu, L. Shi, X. Hu, M. Chen, L. Wu, A structural polymer for highly efficient all-day passive radiative cooling. *Nat. Commun.* **12**, 365 (2021). doi:10.1038/s41467-020-20646-7

12. T. Li, Y. Zhai, S. He, W. Gan, Z. Wei, M. Heidarinejad, D. Dalgo, R. Mi, X. Zhao, J. Song, J. Dai, C. Chen, A. Aili, A. Vellore, A. Martini, R. Yang, J. Srebric, X. Yin, L. Hu, A radiative cooling structural material. *Science* **364**, 760-763 (2019). doi:10.1126/science.aau9101
13. Y. Peng, J. Chen, A. Y. Song, P. B. Catrysse, P.-C. Hsu, L. Cai, B. Liu, Y. Zhu, G. Zhou, D. S. Wu, H. R. Lee, S. Fan, Y. Cui, Nanoporous polyethylene microfibres for large-scale radiative cooling fabric. *Nat. Sustain.* **1**, 105-112 (2018). doi:10.1038/s41893-018-0023-2
14. C. Lin, Y. Li, C. Chi, Y. S. Kwon, J. Huang, Z. Wu, J. Zheng, G. Liu, C. Y. Tso, C. Y. H. Chao, B. Huang, A solution-processed inorganic emitter with high spectral selectivity for efficient subambient radiative cooling in hot humid climates. *Adv. Mater.* **34**, 2109350 (2022). doi:10.1002/adma.202109350
15. X. Xue, M. Qiu, Y. Li, Q. M. Zhang, S. Li, Z. Yang, C. Feng, W. Zhang, J.-G. Dai, D. Lei, W. Jin, L. Xu, T. Zhang, J. Qin, H. Wang, S. Fan, Creating an eco-friendly building coating with smart subambient radiative cooling. *Adv. Mater.* **32**, 1906751 (2020). doi:10.1002/adma.201906751
16. Y. Tian, H. Shao, X. Liu, F. Chen, Y. Li, C. Tang, Y. Zheng, Superhydrophobic and recyclable cellulose-fiber-based composites for high-efficiency passive radiative cooling. *ACS Appl. Mater. Interfaces* **13**, 22521-22530 (2021). doi:10.1021/acsami.1c04046
17. S. Wang, Y. Wang, Y. Zou, G. Chen, J. Ouyang, D. Jia, Y. Zhou, Biologically inspired scalable-manufactured dual-layer coating with a hierarchical micropattern for highly efficient passive radiative cooling and robust superhydrophobicity. *ACS Appl. Mater. Interfaces* **13**, 21888-21897 (2021). doi:10.1021/acsami.1c05651
