# Supplementary material for: Durable radiative cooling against environmental aging
Source: Nat Commun. 2022 Aug 16;13:4805. doi: 10.1038/s41467-022-32409-7 (PMC9381728; doi:10.1038/s41467-022-32409-7)
Supplement: Supplementary file 2 — Description of Additional Supplementary Files [file 41467_2022_32409_MOESM2_ESM.pdf]

Description of Additional Supplementary Files

File Name: Supplementary Movie 1
Description: Viscous mud dripped and flowed off the AACP coating.

File Name: Supplementary Movie 2
Description: Cleaning effect of AACP when MnO_2 was soiling agent.

File Name: Supplementary Movie 3
Description: Water jet impact showing AACP coating's mechanical robustness.

File Name: Supplementary Movie 4
Description: Infrared video of AACP coated wall tile showing temperature reduction.

File Name: Supplementary Movie 5
Description: AACP coated wall tile showing excellent anti-soiling function (the tile was tilted at about 20°).
